# Inequalities in Disability-Free and Disabling Multimorbid Life Expectancy in Costa Rica, Mexico, and the United States

**DOI:** 10.1093/geronb/gbae093

**Published:** 2024-05-24

**Authors:** Anastasia Lam, Katherine Keenan, Geneviève Cézard, Hill Kulu, Mikko Myrskylä

**Affiliations:** Max Planck Institute for Demographic Research, Rostock, Germany; School of Geography and Sustainable Development, University of St Andrews, St Andrews, UK; School of Geography and Sustainable Development, University of St Andrews, St Andrews, UK; Department of Public Health and Primary Care, University of Cambridge, Cambridge, UK; School of Geography and Sustainable Development, University of St Andrews, St Andrews, UK; Max Planck Institute for Demographic Research, Rostock, Germany; Center for Social Data Science and Population Research Unit, University of Helsinki, Helsinki, Finland; (Social Sciences Section)

**Keywords:** Aging, Cumulative disadvantage, Disability, Multimorbidity, Multistate modeling

## Abstract

**Objectives:**

To better understand variations in multimorbidity severity over time, we estimate disability-free and disabling multimorbid life expectancy (MMLE), comparing Costa Rica, Mexico, and the United States (US). We also assess MMLE inequalities by sex and education.

**Methods:**

Data come from the Costa Rican Study on Longevity and Healthy Aging (2005–2009), the Mexican Health and Aging Study (2012–2018), and the Health and Retirement Study (2004–2018). We apply an incidence-based multistate Markov approach to estimate disability-free and disabling MMLE and stratify models by sex and education to study within-country heterogeneity. Multimorbidity is defined as a count of 2 or more chronic diseases. Disability is defined using limitations in activities of daily living.

**Results:**

Costa Ricans have the lowest MMLE, followed by Mexicans, then individuals from the US. Individuals from the US spend about twice as long with disability-free multimorbidity compared with individuals from Costa Rica or Mexico. Females generally have longer MMLE than males, with particularly stark differences in disabling MMLE. In the US, higher education was associated with longer disability-free MMLE and shorter disabling MMLE. We identified evidence for cumulative disadvantage in Mexico and the US, where sex differences in MMLE were larger among the lower educated.

**Discussion:**

Substantial sex and educational inequalities in MMLE exist within and between these countries. Estimating disability-free and disabling MMLE reveals another layer of health inequality not captured when examining disability and multimorbidity separately. MMLE is a flexible population health measure that can be used to better understand the aging process across contexts.

Multimorbidity—often defined as the coexistence of two or more chronic diseases—is an increasingly important global public health issue (The Academy of Medical Sciences, 2018). Multimorbidity prevalence has been increasing worldwide and is projected to continue rising as populations age (The Academy of Medical Sciences, 2018). In the growing literature on multimorbidity, there remain notable gaps and weaknesses. Namely, studies tend to focus on estimating prevalence and disease clustering using cross-sectional approaches, whereas longitudinal research on time spent with multimorbidity and multimorbidity severity is limited, especially in low- and middle-income contexts (Cezard et al., 2021). This paper aims to fill these gaps by using an incidence-based multistate Markov approach and longitudinal panel data from Costa Rica, Mexico, and the United States (US) to gain a more nuanced understanding of inequalities in multimorbid life expectancy (MMLE) across contexts.

Multimorbidity is often associated with the development of disability, which in turn affects multimorbidity severity, highlighting the importance to concurrently examine both conditions (Calderón-Larrañaga et al., 2019). However, existing studies on health expectancies have included either multimorbidity or disability and tend to focus on time spent without those conditions (Mehta & Myrskylä, 2017; Payne, 2018, 2022). On the contrary, we focus on time spent with multimorbidity to identify people who are more disadvantaged (i.e., who live longer with disease). MMLE is an aggregate measure that does not capture how certain diseases may be more disabling than others, that is, stroke versus diabetes. We address this by incorporating disability status into MMLE as a proxy to separate multimorbidity into less severe (disability-free) and more severe (disabling) states. By extending this concept to a cross-national comparative framework, we can better examine how the aging process differs at both the micro- and macrolevel, thus identifying where and how inequalities are concentrated.

We chose to compare Costa Rica, Mexico, and the US for several reasons. The three countries are rapidly aging, and the leading causes of death have converged over time (Table 1). Costa Rica and Mexico are both middle-income countries with a similar history of demographic, epidemiological, and nutritional transitions (Atun et al., 2015). Compared to the US, both countries spend less of their GDP on health, but had lower life expectancies (LE) at age 60 in 2019 (about 1 year less in Costa Rica and 3 years less in Mexico). All 3 countries also have nationally representative longitudinal harmonized data. Therefore, extending the comparison beyond mortality to consider the time spent in various states of morbidity would enrich our understanding of health inequalities across the life course and how it plays out in these different settings.

**Table 1.** Economic, Population, and Health Indicators for Costa Rica, Mexico, and the United States

Indicator	Costa Rica			Mexico			United States		
	1950	2000	2019	1950	2000	2019	1950	2000	2019
GDP/capita, (current USD)	381^a^	3,789	12,762	345.2^a^	7,158	9,950	3,007^a^	36,330	65,095
Health expenditure (% of GDP)	—	6.6	7.3	—	4.5	5.4	—	12.5	16.7
LE at birth, male	52.2	75.3	77.0	42.4	70.7	70.9	65.4	74.1	76.6
LE at birth, female	55.5	80.0	82.0	45.6	76.5	77.6	71.0	79.4	81.7
LE at age 60, male	14.3	20.7	21.1	13.2	19.7	19.4	15.7	19.7	22.2
LE at age 60, female	15.5	23.4	24.4	14.0	22.4	22.1	18.6	23.0	25.3
Population size (millions)	1	4	5	28	98	125	148	282	334
% over age 60	4.8	8.5	14.4	4.8	7.3	11.5	12.2	16.2	22.1
Infant mortality rate	114.2	11.2	7.0	159.6	23.4	12.0	31.7	7.2	5.5
Leading causes of death	Infections, malnutrition, CVD, diabetes, cancer	CVD, cancer, digestive diseases	CVD, cancer, diabetes, CKD	Digestive diseases, pneumonia, influenza, congenital deformations	CVD, diabetes, CKD, cancer	CVD, diabetes, CKD, cancer	CVD, cancer, vascular lesions	CVD, cancer, stroke	CVD, cancer, accidents

*Notes:* CKD = chronic kidney disease; CVD = cardiovascular disease; GDP = gross domestic product; LE = life expectancy; USD = United States Dollar.

*Source:* Bastian et al., 2020; Institute for Health Metrics and Evaluation, 2019; Rabell & Terán, 1986; Rosero-Bixby, 1994; United Nations Population Division, Department of Economic and Social Affairs, 2022; World Bank, 2022.

^a^Values in these cells are from 1960 instead of 1950.

Health system differences are pronounced and might contribute to morbidity and mortality disparities. Of these three countries, Costa Rica is the only one with a universal healthcare system that emphasizes comprehensive primary care (Atun et al., 2015). Mexico had a public health insurance scheme (Seguro Popular) that ran from 2003–2020. At its peak, it managed to expand coverage to almost half the population but struggled with funding and meeting the demand for chronic disease services (Knaul et al., 2023). The US has a fragmented public/private system, where public coverage includes Medicare (for adults aged 65 and older) and Medicaid (for low-income adults) (Dickman et al., 2017).

## Life Expectancy Trends

Despite the US undergoing the epidemiological transition earlier, Costa Rica and Mexico quickly narrowed the gap. The rapid LE gains in Costa Rica and Mexico from 1950 to 2000 can be explained by significant reductions in infant mortality, infectious disease mortality, and more recently, cardiovascular mortality (Table 1). In a comparative study of Costa Rica, Mexico, Puerto Rico, and the US, LE and proportion of LE spent disability-free at age 65 were similar across countries, except that Costa Rican females spent slightly more years with disability than females in other countries (Payne, 2018). Another study, using a combined education and income relative scale, found higher LE in the US than in Costa Rica among people of the highest socioeconomic quartile, but lower LE for those of the lowest socioeconomic quartile (Rosero-Bixby & Dow, 2016). These contrasting findings were attributed to significantly higher lung cancer and heart disease mortality rates and greater socioeconomic inequalities in the US (Rosero-Bixby & Dow, 2016).

## Aging, Multimorbidity, and Disability

The percentage of the population older than 60 years in each country has been increasing substantially over time, up to 11.5% in Mexico, 14.4% in Costa Rica, and 22.1% in the US in 2019 (Table 1). With this comes increased chronic disease prevalence, and subsequently multimorbidity and disability. In Costa Rica, studies on multimorbidity and disability are limited. A longitudinal study estimated that 12% of older adults suffer from multimorbidity and 19% from disability (defined using activities of daily living [ADLs] and instrumental ADLs) (Vazquez-Castillo, 2023). In Mexico, multimorbidity prevalence in adults aged 50+ ranges from 27% to 35% (Islas-Granillo et al., 2018; Rivera-Almaraz et al., 2018). Having multimorbidity makes having disability more likely, and the time spent with ADL disability is increasing over time (Payne & Wong, 2019). In the US, the prevalence of multimorbidity in older adults ranges from about 59% to 81% and increases with age (Boersma et al., 2020; Buttorff et al., 2017). Although the time spent with chronic morbidities has increased across birth cohorts, the time with ADL disability has remained relatively stable (Payne, 2022). Unlike disability, defined using ADLs in the aforementioned studies, multimorbidity is often measured differently across studies, making comparisons difficult (Ho et al., 2021). We overcome this lack of standardization by using the same approach and including the same list of conditions to define multimorbidity across each data source to facilitate comparisons.

## Health Inequalities by Sex and Education

Globally, females have higher rates of multimorbidity than males (Garin et al., 2016). Compared to males, females seek care earlier and more often, so have higher rates of diagnosis but could also be more biologically susceptible to certain diseases (Höhn et al., 2020). Females also usually live longer than males, but in poorer health and with more disability. Studies suggest that females have a higher prevalence of chronic diseases than males in our three countries (Buttorff et al., 2017; Garin et al., 2016; Harhay et al., 2016; Santamaría-Ulloa et al., 2019). A Mexican study also found increasing sex differences over time; from 2001 to 2018, the difference in multimorbidity prevalence between females and males rose from 11% to 18% (Rojas-Huerta et al., 2022).

By contrast, the relationship between education and multimorbidity is inconsistent across contexts. This could be due to heterogeneity in the measurement of multimorbidity or varying associations between education and certain diseases. We did not find studies investigating education and multimorbidity in Costa Rica, but found that lower levels of education are associated with a higher likelihood of chronic kidney disease and hypertension, whereas higher levels of education are associated with more obesity (Harhay et al., 2016; Rehkopf et al., 2010). In Mexico, one study found higher multimorbidity prevalence in people with lower levels of education (Macinko et al., 2019), whereas another study found no association (Islas-Granillo et al., 2018). In the US, lower levels of education are associated with a higher likelihood of multimorbidity (Chamberlain et al., 2020; Johnson-Lawrence et al., 2017).

## Cumulative (Dis)advantage

The cumulative (dis)advantage hypothesis posits that social and structural systems shape how individuals accumulate advantages or disadvantages over time (Dannefer, 2003). Multimorbidity can be thought of as a biosocial process of cumulative disadvantage where diseases accumulate over the life course. The extent of disadvantage can vary within and between countries due to differences in social, demographic, and structural factors, as well as inequalities in access to health and social care. Although cumulative (dis)advantage has mainly been used to understand disparities within a single setting, recent studies have taken a comparative approach to understand how differences in political, educational, and health systems might shape the cumulative (dis)advantage process across countries (Leopold, 2018; Wetzel & Vanhoutte, 2020). However, these studies have only focused on the US and European contexts. We do not know what the process looks like in low- and middle-income countries and how that compares to high-income countries. Therefore, we use cumulative (dis)advantage as a framework to understand how inequalities in MMLE by sex and education differ within and between Costa Rica, Mexico, and the US.

## Method

### Data

Data are from Waves 1–3 (2005–2009) of the Costa Rican Study on Longevity and Healthy Aging (CRELES) Pre-1945 Cohort (Rosero-Bixby et al., 2013), Waves 3–5 (2012–2018) of the Mexican Health and Aging Study (MHAS) (Wong et al., 2017), and Waves 7–14 (2004–2018) of the Health and Retirement Study (HRS) (RAND, 2022). Harmonized data were obtained through the Gateway to Global Aging Data (g2aging.org). CRELES and MHAS were modeled after the HRS, which allows for data harmonization and promotes cross-country comparisons.

CRELES recruited participants aged 60 and older, with an oversampling of older ages, and followed participants every 2 years (Rosero-Bixby et al., 2013). MHAS recruited participants aged 50 and older, and their spouses regardless of age (Wong et al., 2017). Waves 1 and 2 of MHAS occurred in 2001 and 2003, but Wave 3 did not take place until 2012, and subsequent waves occurred every 3 years. Our method requires evenly spaced time intervals between waves, which is why Wave 3 is the baseline (see *Multistate Modeling Approach* section for further details). HRS surveyed individuals from age 50, and their spouses regardless of age, every 2 years from 1992 (Sonnega et al., 2014). We took Wave 7 as our baseline to align the time period with the other studies.

We included proxy respondents in our study for various reasons. A previous study that used CRELES excluded proxy respondents because they tended to be older and had lower LE than the self-respondents, which would bias the overall LE (Rueda-Salazar et al., 2021). In our case, however, because we were interested in time spent in ill health, it was important to ensure that the oldest and more impaired/ill participants were also included. Additionally, excluding proxy respondents would have made our CRELES sample too small to feasibly conduct this analysis. Although this might bias the overall LE we estimated, it should provide a more accurate picture of MMLE, particularly at the oldest ages.

The initial sample of CRELES, MHAS, and HRS included 2,798, 21,704, and 32,968 participants, respectively (Supplementary Material Section I). We included participants aged 60 or older, with at least one transition (i.e., being present for more than one wave or dying after one wave), and with sufficient health and sociodemographic information. MHAS and HRS participants who were initially under age 60 became eligible for inclusion once they turned 60 years old and met the other inclusion criteria. Exclusions for MHAS and HRS were mainly due to participants being present in only one wave (MHAS: 31%, HRS: 6%) or being younger than age 60 (MHAS: 17%, HRS: 26%). The high proportion of single-wave participants in MHAS is because MHAS added a refreshment sample of 4,809 individuals in the 2018 wave. More detailed reasons and number of participants excluded can be seen in Supplementary Material Section I. The final sample sizes were *n* = 2,626 in CRELES, *n* = 11,208 in MHAS, and *n* = 22,345 in HRS.

The rate of attrition between waves, due to death, loss to follow-up, or refusal, was about 9% in CRELES, 6%–17% in MHAS, and 7%–18% in the HRS. To compare the included and excluded samples, we provide descriptive information of excluded participants who were aged 60 or older. The average age of excluded participants from MHAS and HRS was 6.8 and 9.3 years younger than that of included participants, respectively. Excluded participants from all three surveys were more highly educated than included participants. Compared to included participants, excluded CRELES participants had lower initial prevalence of no disease, excluded MHAS participants had lower initial prevalence of multimorbidity, and excluded HRS participants had lower initial prevalence of disabling multimorbidity.

### Measures

We define multimorbidity as concurrently having two or more of the following diseases: arthritis, cancer, diabetes, heart problems (including heart attack), hypertension, stroke, and respiratory problems. These diseases were chosen because they were common across the surveys and are also among the leading causes of morbidity and mortality in the region (Vos et al., 2020). A disease was indicated as present if the participant reported ever having been told by a doctor that they had that disease. All diseases were defined as being chronic and irreversible for the purposes of this analysis. Each survey asked about different ADLs, so we created a composite variable using the ADLs included across all three surveys: eating, bathing, walking, and getting in and out of bed. Therefore, additional ADLs, such as dressing and toileting, were not included. These ADLs were used to define whether someone was disability-free (no difficulty with any ADL) or had a disability (some difficulty with at least one ADL). Disability may be reversible, or at least improved, but this usually requires some form of intervention (Szanton et al., 2021). Therefore, for this study, we did not account for reversals in disability status. Mortality information was obtained through next-of-kin or surviving family interviews for CRELES and MHAS, and through relatives or the National Death Index for HRS. Sex was categorized as “male” or “female.” We defined education as the highest level of completed education, which was categorized into the following levels: “primary school or less,” “secondary school,” and “postsecondary school.”

#### Measuring cumulative (dis)advantage

We follow the method for identifying cumulative (dis)advantage proposed by Hale et al. (2022). Because of our focus on multimorbidity, where the literature suggests that females and low-educated groups are likely to be disadvantaged (i.e., have more multimorbidity), we assume that being male and higher educated results in lower MMLE. To determine whether there is evidence for cumulative (dis)advantage within each country, we subtract the male MMLE from the female MMLE within the low-education group (primary school or less) and the high-education group (postsecondary school). If the sex difference in MMLE is larger in the low-education group than in the high-education group, this indicates that low-educated females experience cumulative *disadvantage*. If the sex difference is larger in the high-education group than in the low-education group, then high-educated males experience cumulative *advantage*. To examine how cumulative (dis)advantage differs between countries, we compare the sex difference within and between education groups. A larger sex difference within education groups indicates greater sex inequalities in that country, while a larger sex difference between education groups indicates greater educational inequalities.

### Statistical Analysis

We obtained descriptive statistics stratified by sex for each country for age, education, origin disease states, deaths, person-years of follow-up, and number of transitions between states. We also calculated the prevalence of disease at one’s origin state for those aged 60–69.

#### Multistate modeling approach

**Figure 1.**
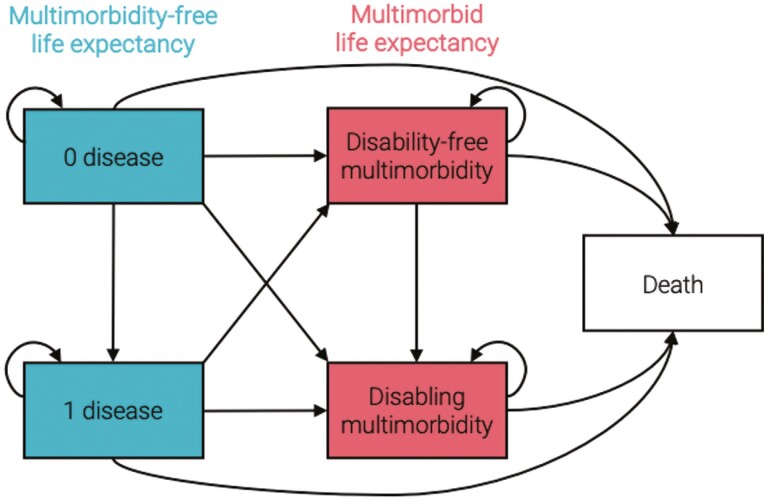
State space showing all possible transitions to and from different disease states and death. All arrows point in one direction, indicating that transitions can only occur in that direction. Curved arrows pointing back to the same state indicate remaining in that state. Multimorbidity-free life expectancy encompasses the no disease and one disease states and multimorbid life expectancy encompasses the disability-free and disabling multimorbidity states.

Multinomial logit models for each country, adjusted for age, stratified by sex and/or education, and weighted using sampling weights from each survey, were used to compute transition probabilities. These probabilities estimate transitions between the following states: no disease, one disease, disability-free multimorbidity, disabling multimorbidity, and death (Figure 1). Individuals can remain in the same state throughout the study period, transition to a subsequent state, or die. Death is an absorbing state, meaning once someone enters that state, they cannot leave.

These transition probabilities are input into discrete-time multistate Markov models to estimate state expectancies and LE using the standard approach (Schneider et al., 2023). Discrete-time multistate Markov models are less biased than the Sullivan Method when incidence and mortality rates change over time (Barendregt et al., 1994), and they enable us to capitalize on panel data to model transitions into and out of different states (Lynch & Brown, 2010). The Sullivan Method combines age-specific prevalence estimates with period life table and splits the years lived in the life table into healthy and unhealthy, proportional to prevalence (Sullivan, 1971). The standard approach we use involves computing expectancies conditional on an initial age of 60 and then obtaining a weighted average across these values. The weights correspond to the distribution of individuals in each state at age 60, but to account for small sample sizes, we take the average distribution for ages 60–69. This method requires the time intervals between survey waves to be evenly spaced (Schneider et al., 2023), and in this case the time between waves is 2 years (CRELES and HRS) or 3 years (MHAS). As long as the age grids used to estimate the transition probabilities match the spacing between survey waves, the expectancy estimates across data sets can be compared even if the time intervals differ. We computed 95% confidence intervals based on asymptotic theory and the delta method (Schneider, 2023). The delta method approach does not restrict confidence limits, which allows negative confidence limits to be produced. Because negative expectancies are impossible, the limit was set equal to zero if negative values were present. The underlying variance-covariance matrix of the multinomial logit model accounts for the complex survey designs for each data set.

### Sensitivity Analyses

We conducted a sensitivity analysis excluding hypertension from the definition of multimorbidity because there is debate about whether it is a disease or only a risk factor (The Lancet, 2019). However, hypertension was included in the definition for 316 (70%) of 452 studies on multimorbidity (Ho et al., 2021). We hypothesize that fewer participants will have multimorbidity once hypertension is excluded, but the general patterns for MMLE should remain consistent.

Statistical analyses were conducted in Stata 17 (StataCorp, 2021) and figures were created in R version 4.2.1 (R Core Team, 2020). Expectancy estimates and confidence intervals were obtained using the *dtms* package (Schneider, 2023).

## Results

### Descriptive Statistics

Table 2 provides descriptive characteristics of the samples by country and sex. At entry wave, participants from Costa Rica had the highest average age (76.9 years, *SD* 10.3), followed by those from Mexico (70.0 years, *SD* 8.0), and the US (68.9 years, *SD* 8.9). This difference is likely due to the oversampling of the oldest ages in CRELES. People from the US are the highest educated, with 94% having at least secondary education. In contrast, most participants from Costa Rica and Mexico have only a primary school education or less (87% and 76%, respectively). In the US, 47% of participants enter the study with disability-free multimorbidity and 14% enter with disabling multimorbidity, which are greater than in both Mexico (33% and 11%, respectively) and Costa Rica (25% and 9%, respectively). Females have a higher prevalence of multimorbidity than males, except for males from the US who have a higher prevalence of disability-free multimorbidity than US females. In the US, the proportion who died throughout the study period is twice as high as in the other two countries (39% vs 20% in Costa Rica and 18% in Mexico), likely because the follow-up period was twice as long. The percentage of participants remaining in the same disease state ranges from 24.6% to 60.1%, and the most common transition is from disabling multimorbidity to death (5.5%–11.7%). Information on disease prevalence can be seen in Supplementary Material Section II.

**Table 2.** Unweighted Descriptive Statistics of Participants’ Age, Education, and Disease States Are Based on Their First Wave, Whereas Deaths, Person-Years of Follow-up, and Transitions Are Based on Total Follow-Up Time

Characteristic	Costa Rica		Mexico		United States	
	Male (*n* = 1,200)	Female (*n* = 1,426)	Male (*n* = 4,994)	Female (*n* = 6,214)	Male (*n* = 9,599)	Female (*n* = 12,746)
Mean age at entry (*SD*)	76.9 (10.3)	76.9 (10.2)	70.2 (7.9)	69.9 (8.1)	68.6 (8.3)	69.2 (9.2)
Educational attainment, *n* (%)						
Primary school or less	1,040 (86.7%)	1,248 (87.5%)	3,691 (73.9%)	4,868 (78.3%)	680 (7.1%)	776 (6.1%)
Secondary school	93 (7.7%)	107 (7.5%)	784 (15.7%)	1,075 (17.3%)	4,540 (47.3%)	6,969 (54.7%)
Post-secondary school	67 (5.6%)	71 (5.0%)	519 (10.4%)	271 (4.4%)	4,379 (45.6%)	5,001 (39.2%)
Origin disease state, *n* (%)						
0 disease	438 (36.5%)	332 (23.3%)	1,607 (32.2%)	1,048 (16.9%)	1,503 (15.7%)	1,625 (12.7%)
1 disease	436 (36.3%)	525 (36.8%)	1,662 (33.3%)	1,973 (31.8%)	2,457 (25.6%)	3,259 (25.6%)
Disability-free multimorbidity	237 (19.8%)	413 (29.0%)	1,337 (26.8%)	2,333 (37.5%)	4,614 (48.1%)	5,871 (46.1%)
Disabling multimorbidity	89 (7.4%)	156 (10.9%)	388 (7.8%)	860 (13.8%)	1,025 (10.7%)	1,991 (15.6%)
Disability at origin disease state, *n* (%)	216 (22.0%)	341 (31.5%)	664 (15.3%)	1,229 (24.7%)	1,338 (16.2%)	2,515 (24.6%)
0 disease	52 (24.1%)	73 (21.4%)	98 (14.8%)	91 (7.4%)	67 (5.0%)	76 (3.0%)
1 disease	75 (34.7%)	112 (32.8%)	178 (26.8%)	278 (22.6%)	182 (13.6%)	353 (14.0%)
Multimorbidity	89 (41.2%)	156 (45.7%)	388 (58.4%)	860 (70.0%)	1,089 (81.4%)	2,086 (82.9%)
Deaths, *n*	253 (21.1%)	264 (18.5%)	999 (20.0%)	1,015 (16.3%)	4,002 (41.7%)	4,693 (36.8%)
Person-years of follow-up, *n*	6,212	7,470	24,892	31,222	96,420	132,964
Transitions, *n* (%)	3,106	3,735	12,446	15,611	48,210	66,482
0 disease to 0 disease	894 (47.1%)	639 (33.6%)	3,130 (49.4%)	1,990 (31.4%)	3,843 (36.8%)	4,317 (41.4%)
0 disease to 1 disease	101 (5.3%)	94 (4.9%)	421 (6.6%)	320 (5.0%)	752 (7.2%)	858 (8.2%)
0 disease to disability-free MM	17 (0.9%)	7 (0.4%)	68 (1.1%)	60 (0.9%)	176 (1.7%)	151 (1.4%)
0 disease to disabling MM	4 (0.2%)	6 (0.3%)	21 (0.3%)	17 (0.3%)	38 (0.4%)	33 (0.3%)
0 disease to death	78 (4.1%)	59 (3.1%)	241 (3.4%)	97 (1.5%)	155 (1.5%)	109 (1.0%)
1 disease to 1 disease	903 (35.9%)	1,140 (45.3%)	3,266 (36.3%)	3,857 (42.9%)	7,443 (32.1%)	10,447 (45.1%)
1 disease to disability-free MM	101 (4.0%)	90 (3.6%)	458 (5.1%)	474 (5.3%)	1,636 (7.1%)	2,000 (8.6%)
1 disease to disabling MM	45 (1.8%)	66 (2.6%)	142 (1.6%)	213 (2.4%)	268 (1.2%)	457 (2.0%)
1 disease to death	87 (3.5%)	86 (3.4%)	288 (3.2%)	288 (3.2%)	436 (1.9%)	466 (2.0%)
Disability-free MM to disability-free MM	540 (30.5%)	911 (51.4%)	2,856 (30.3%)	5,028 (53.4%)	22,900 (38.8%)	28,694 (48.7%)
Disability-free MM to disabling MM	73 (4.1%)	154 (8.7%)	270 (2.9%)	647 (6.9%)	1,495 (2.5%)	2,555 (4.3%)
Disability-free MM to death	40 (2.3%)	53 (3.0%)	315 (3.3%)	308 (3.3%)	1,796 (3.0%)	1,525 (2.6%)
Disabling MM to disabling MM	175 (26.8%)	364 (55.7%)	815 (24.6%)	1,990 (60.1%)	5,657 (25.5%)	12,277 (55.4%)
Disabling MM to death	48 (7.4%)	66 (10.1%)	182 (5.5%)	322 (9.7%)	1,615 (7.3%)	2,593 (11.7%)

*Notes:* MM = Multimorbidity; *SD* = Standard deviation.

Data are from the Costa Rican Study on Longevity and Health Aging, the Mexican Health and Aging Study, and the Health and Retirement Study.

### Transition Probabilities

**Figure 2.**
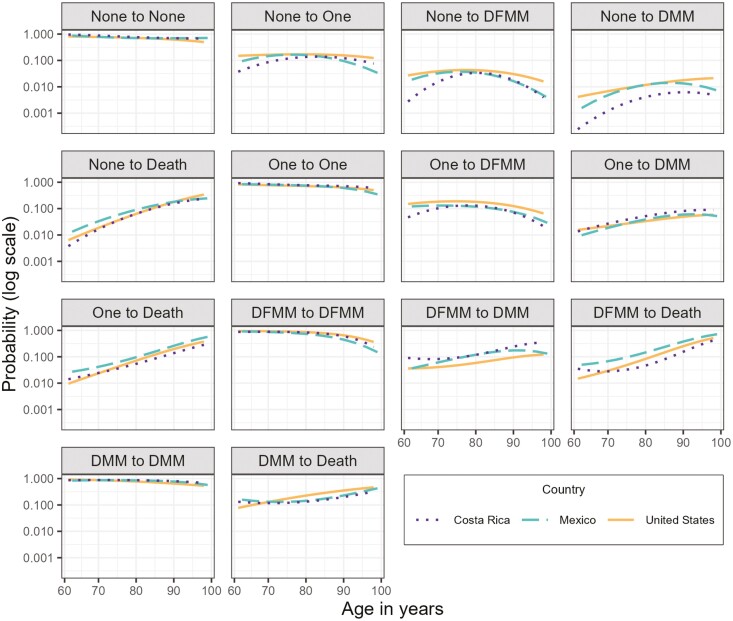
Probability of transitioning between disease states from age 60-100 for males in Costa Rica, Mexico, and the United States. DFMM = disability-free multimorbidity; DMM = disabling multimorbidity; None = no disease; One = one disease.

Figure 2 shows transition probabilities for males. Probabilities for females are similar and can be seen in Supplementary Material Section III. Costa Ricans are most likely to transition from one disease or disability-free multimorbidity to disabling multimorbidity. Mexican males have the highest probability of transitioning to death from one disease and disability-free multimorbidity, whereas Mexican females have the highest transition probability to death from one disease. Individuals from the US have the highest probability of transitioning to death from disabling multimorbidity. Individuals from the US also tend to have the highest probabilities of transitioning from zero disease to subsequent states and from one disease to disability-free multimorbidity. The patterns for males and females are generally similar, but the biggest differences can be seen for the transitions from zero and one disease.

### Life Expectancy

Average remaining LE for males at age 60 is 24.3 years (95% CI: 22.9–25.8) in Costa Rica, 22.9 years (95% CI: 21.8–24.0) in Mexico, and 20.8 years (95% CI: 20.5–21.2) in the US (Table 3). For females, remaining LE is 25.1 years (95% CI: 23.3–26.9) in Costa Rica, 25.4 years (95% CI: 24.4–26.3) in Mexico, and 23.1 years (95% CI: 22.8–23.4) in the US. More detailed life and state expectancy estimates can be found in Supplementary Material Section IV. Our LE estimates for Costa Rican males and Mexican males and females are higher than WHO estimates by 1.9–2.5 years and UN estimates by 3.0–3.2 years (Costa Rican males 2005, WHO: 22.4, UN: 21.3; Mexican males 2015, WHO: 20.4, UN: 19.7; Mexican females 2015, WHO: 22.9, UN: 22.3; Supplementary Material Section V). However, they are closer to LE estimates provided by other studies. For the US, our LE estimate for males is very similar to vital statistics, but our estimate for females is lower by 1–1.5 years. The discrepancies we observe may be attributable to differences in study periods, study samples being healthier than the general population, and/or varying methods of estimating LE, which would produce different estimates (Murakami et al., 2018).

**Table 3.** Average Expectancies at Age 60, by Country and Sex, Based on Data From the Costa Rican Study on Longevity and Health Aging, the Mexican Health and Aging Study, and the Health and Retirement Study

Country	Sex	Destination state	Average expectancy	Lower 95% CI	Upper 95% CI	% of total life expectancy^a^
Costa Rica	Male	0 disease	6.2	5.5	7.0	25.6
		1 disease	6.6	5.6	7.5	26.9
		Disability-free multimorbidity	6.0	5.1	6.9	24.8
		Disabling multimorbidity	5.5	4.2	6.9	22.7
		Multimorbidity-free life expectancy	12.8	11.1	14.5	52.5
		Multimorbid life expectancy	11.5	9.3	13.8	47.5
		Life expectancy	24.3	22.9	25.8	100.0
	Female	0 disease	2.5	1.9	3.0	9.9
		1 disease	7.5	6.6	8.5	29.9
		Disability-free multimorbidity	6.6	5.7	7.4	26.1
		Disabling multimorbidity	8.6	7.2	10.0	34.2
		Multimorbidity-free life expectancy	10.0	8.5	11.5	39.8
		Multimorbid life expectancy	15.2	12.9	17.4	60.2
		Life expectancy	25.1	23.3	26.9	100.0
Mexico	Male	0 disease	4.9	4.4	5.5	21.4
		1 disease	6.6	5.8	7.3	28.7
		Disability-free multimorbidity	7.0	6.2	7.7	30.5
		Disabling multimorbidity	4.4	3.6	5.3	19.4
		Multimorbidity-free life expectancy	11.5	10.2	12.8	50.1
		Multimorbid life expectancy	11.4	9.8	13	49.9
		Life expectancy	22.9	21.8	24	100.0
	Female	0 disease	2.5	2.3	2.8	9.9
		1 disease	5.6	5.2	6.1	22.1
		Disability-free multimorbidity	9.3	8.6	9.9	36.6
		Disabling multimorbidity	7.9	7.0	8.9	31.3
		Multimorbidity-free life expectancy	8.1	7.5	8.9	32.1
		Multimorbid life expectancy	17.2	15.6	18.8	67.9
		Life expectancy	25.4	24.4	26.3	100.0
United States	Male	0 disease	1.4	1.3	1.5	6.8
		1 disease	3.2	3.0	3.3	15.3
		Disability-free multimorbidity	11.9	11.6	12.2	57.0
		Disabling multimorbidity	4.4	4.1	4.6	20.9
		Multimorbidity-free life expectancy	4.6	4.3	4.8	22.1
		Multimorbid life expectancy	16.3	15.7	16.8	77.9
		Life expectancy	20.8	20.5	21.2	100.0
	Female	0 disease	1.3	1.2	1.4	5.6
		1 disease	3.3	3.1	3.4	14.1
		Disability-free multimorbidity	11.1	10.9	11.4	48.1
		Disabling multimorbidity	7.4	7.2	7.7	32.2
		Multimorbidity-free life expectancy	4.6	4.3	4.8	19.7
		Multimorbid life expectancy	18.5	18.1	19.1	80.3
		Total life expectancy	23.1	22.8	23.4	100.0

*Notes:* CI = Confidence interval.

^a^The percent of total life expectancy calculated here is based on unrounded average expectancies and thus may differ slightly from percentages based on the rounded average expectancies presented in the table.

### Multimorbid Life Expectancy

To provide a comprehensive overview of LE with and without multimorbidity, we start by briefly presenting results for multimorbidity-free LE (the sum of remaining LE with 0 or 1 disease), before focusing on MMLE. Costa Ricans have the highest multimorbidity-free LE, followed closely by Mexicans (Table 3). In the US, multimorbidity-free LE is about one-third to half the multimorbidity-free LE of Costa Rica and Mexico. In Costa Rica and Mexico, males have higher multimorbidity-free LE than females, while in the US there is no sex difference.

MMLE for males in Costa Rica, Mexico, and the US is 11.5 years, 11.4 years, and 16.3 years, respectively (Table 3). This translates into the percentage of LE spent with multimorbidity being 47% in Costa Rica, 50% in Mexico, and 78% in the US. For females, total MMLE is 15.2 years in Costa Rica, 17.2 years in Mexico, and 18.5 years in the US; the percentage of LE with multimorbidity is 61%, 68%, and 80%, respectively.

If we consider multimorbidity-free LE and MMLE in relation to LE, we generally see that regardless of country, sex, or education, as LE increases, multimorbidity-free LE increases and MMLE decreases (Supplementary Material Section VI). Table 3 shows that this pattern is present for both absolute and relative estimates by sex. However, the US, which has the lowest LE, consistently has the lowest multimorbidity-free LE and highest MMLE. The relationship between MMLE and education varies by sex. Postsecondary educated males have similar or lower multimorbidity-free LE and higher MMLE compared to the lower educated groups. Opposite patterns are seen for females; postsecondary educated females have similar or higher multimorbidity-free LE and similar or lower MMLE compared to lower educated groups.

**Figure 3.**
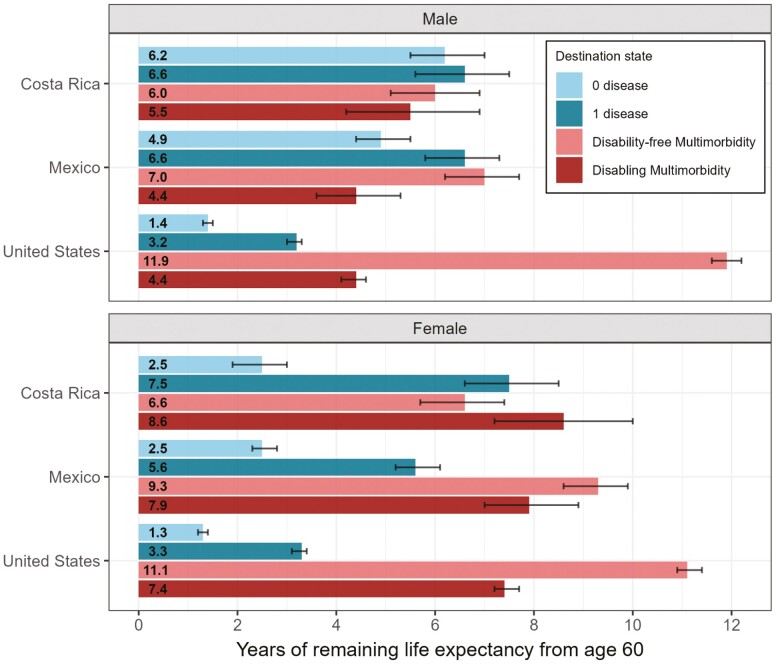
Years of remaining life expectancy spent in each destination state from age 60, by sex and country. The time spent with 0 disease and 1 disease sum up to multimorbidity-free life expectancy and the time spent with disability-free multimorbidity and disabling multimorbidity sum up to multimorbid life expectancy.

When looking at MMLE by disability status, patterns in disability-free MMLE are similar to those observed for MMLE (Figure 3). Costa Ricans have the lowest disability-free MMLE (Males: 6 years, Females: 6.6 years), followed closely by Mexicans (Males: 7 years, Females: 9.3 years). Disability-free MMLE in the US is almost twice that in Costa Rica (Males: 11.9 years, Females: 11.1 years). Conversely, disabling MMLE is lowest in the US (Males: 4.4 years, Females: 7.4 years), with Mexico having similar estimates (Males: 4.4 years, Females: 7.9 years), but Costa Rica has higher disabling MMLE (Males: 5.5 years, Females: 8.6 years).

#### Sex disparities in multimorbid life expectancy

Across the three countries, females have higher MMLE and spend significantly less time with no disease than males (Figure 3). This is particularly apparent for females from Costa Rica and Mexico, who seem to accumulate disease earlier and spend more time with disease than their male counterparts. The greatest sex difference occurs in Mexico, with females having 5.8 more years (18 percentage points) of MMLE than males, compared with 3.7 years (13 percentage points) in Costa Rica and 2.2 years (2 percentage points) in the US. There are no sex differences in disability-free MMLE in Costa Rica. In Mexico and the US, females have 2.3 years and almost 1 year more disability-free MMLE than males, respectively. Females consistently have about 3 years more disabling MMLE than males across all three countries.

#### Educational disparities in multimorbid life expectancy

**Figure 4.**
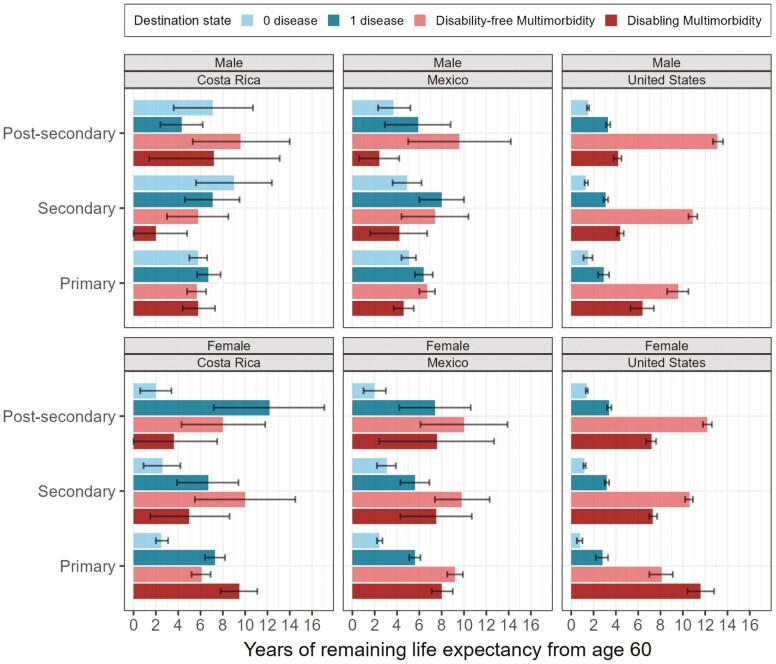
Years of remaining life expectancy spent in each destination state from age 60, by sex, country, and education. The time spent with 0 disease and 1 disease sum up to multimorbidity-free life expectancy and the time spent with disability-free multimorbidity and disabling multimorbidity sum up to multimorbid life expectancy.

In the US, disability-free MMLE increases with higher levels of education, whereas disabling MMLE decreases (Figure 4). The results for Costa Rica and Mexico are rather imprecise and should be interpreted cautiously. In Mexico, we observe similar but weaker patterns for males than we see in the US, but for females, disability-free and disabling MMLE seem stable across education levels. In Costa Rica, disability-free MMLE increases with higher levels of education in males and disabling MMLE decreases with higher education in females.

#### Evidence for cumulative (dis)advantage

We expected to find evidence for cumulative disadvantage within each country, whereby the sex difference (females minus males) in MMLE is larger in the low-education group than in the high-education group. This was true for Mexico and the US, but not for Costa Rica, where there was no clear pattern. In Mexico, the sex difference in MMLE was 5.9 among the low-educated and 5.6 among the high-educated (Supplementary Material Section VII). In the US, the difference was 3.7 among the low-educated and 2.1 among the high-educated.

We also expected that sex and education inequalities in MMLE would be highest in Mexico and the US, respectively, based on inequalities identified in previous studies (Rosero-Bixby, 2018; Rosero-Bixby & Dow, 2016). Within both education groups, the sex difference is larger in Mexico than in the US or Costa Rica (Supplementary Material Section VII). Contrary to our hypothesis, Costa Rica has the largest educational inequalities, whereby the sex differences in the low and high education groups are 4.1 and −5.2, respectively, showing that high-educated females have lower MMLE than high-educated males. This could be a sample size issue due to the small numbers of high educated Costa Ricans in our sample.

### Sensitivity Analysis

When hypertension was excluded from the multimorbidity definition, general patterns remained the same as the main analysis, but multimorbidity-free LE increased, MMLE decreased, and LE stayed about the same (Supplementary Material Section VIII). The largest shift occurred in Costa Rica, where people gained about 5 more years with no disease compared to people in Mexico (4 years) and the US (just over 1 year). Multimorbidity-free LE gains and MMLE losses were similar in Costa Rica and Mexico (6–7 years), whereas it was around 4 years in the US.

## Discussion

In this article, we used three nationally representative surveys from Costa Rica, Mexico, and the US and a discrete-time multistate modeling approach to jointly estimate the time spent in various (multi)morbidity states across the life course, considering sex and education disparities. We incorporate disability status into our measure of MMLE to gain a more nuanced view of multimorbidity severity. We found that regardless of sex or education, people in Costa Rica generally lived longer, healthier lives than people in Mexico and the US, corroborating previous studies and vital statistics (Payne, 2018; World Health Organization, 2020). We observed the greatest differences in disability-free MMLE across countries, with people in the US having almost twice the disability-free MMLE compared to people in Costa Rica. A study that estimated disability-free LE found similar estimates across these three countries (Payne, 2018), which suggests that the cross-country differences we observe may be driven by inequalities in multimorbidity rather than disability. Within all countries, females had higher LE and MMLE than males, with particularly stark differences for disabling MMLE. Although we saw that higher education was associated with longer disability-free MMLE and shorter disabling MMLE in the US, educational gradients in Costa Rica and Mexico were inconclusive. These findings for sex and education inequalities are consistent with those reported in a recent US study (Shen & Payne, 2023).

Using a method that accounts for intersectional sex and educational disadvantages, we identified cumulative disadvantage for MMLE in the US and Mexico. Mexico has the largest sex difference in MMLE, which is similar for both education groups. This could suggest that higher education does not offset sex inequalities in Mexico. Conversely, in the US, the high-education group has a larger decrease in the sex difference, and in Costa Rica the sex difference is completely flipped so that high educated males have higher MMLE than females. These findings demonstrate inequalities in access to education by sex across countries but also indicate that there are context-specific and life-course factors, which play major roles in determining MMLE inequalities. For example, the US has higher lung cancer and heart disease mortality than Costa Rica and higher rates of disability than Mexico, both of which are likely related to the higher prevalence of smoking, obesity, and uncontrolled hypertension, and insufficient health insurance in the US (Gerst-Emerson et al., 2015; Rosero-Bixby & Dow, 2016), which also vary by sex. These poorer health behaviors coupled with differential access to healthcare lead to health disadvantages that can accumulate over the life course (Leopold, 2018; Lu & Shelley, 2019). These health disadvantages are also often strongly associated with socioeconomic disadvantages, which can further contribute to increasing inequalities over time. Future research should focus on trying to better understand the role of these factors and how they shape the profile of multimorbidity, especially within the context of sex and education.

Hypertension and diabetes are some conditions affected by different screening programs across countries, which could lead to substantial variations in diagnoses, prevalence estimates, and lower levels of control in one country versus another (Geldsetzer et al., 2019; Rahim et al., 2023). For example, Costa Rica has the highest proportions of diagnosed and controlled hypertension (81.7% and 53.5%, respectively), compared to 78.4% and 44.8% in the US and 67.5% and 33.7% in Mexico, respectively (NCD Risk Factor Collaboration, 2021). The effect of these differences on estimates of multimorbidity is unknown, but our sensitivity analysis suggests that not accounting for hypertension in multimorbidity drastically shifts the distribution of time spent in each state toward less disease. It would be beneficial for future research to evaluate the association between screening/diagnosis programs and multimorbidity to identify the extent of underestimation.

The people who survived to older ages with multimorbidity, particularly disabling multimorbidity, have accumulated disadvantage throughout their lives in terms of disease and disability. However, other aspects of their lives, such as their educational attainment, resilience, or selection, may give certain individuals an advantage compared to others, both within and between countries (Ben-Shlomo et al., 2016). Our result that Costa Ricans have the greatest disabling MMLE but also generally the longest LE could indicate they are somehow more resilient than their counterparts that their survival selection was stronger, or there are other stronger determinants at play. For example, Costa Rica provides its citizens with integrated public health and primary care services using multidisciplinary medical teams, resulting in high-quality, cost-effective, and equitable care (Pesec et al., 2017). These resources, combined with the high prevalence of low-fatality, disabling diseases (e.g., hypertension, diabetes, arthritis), could explain our finding.

Although we could not account for the role of individual diseases in our estimates, incorporating disability status into MMLE helps us gain insight into the relationship between multimorbidity severity and mortality. It highlights substantial differences that are not captured when looking at multimorbidity and disability separately, and lays the foundation for future studies to delve deeper into this relationship. Additionally, using incidence-based multistate models accounting for the age and health status of an individual and how those change over time enables us to better understand how the social and health processes underlying current morbidity and mortality conditions could influence future circumstances (Saito et al., 2014).

This study has several limitations. First, we used self-reported longitudinal survey data, which is prone to recall bias, survival bias, and attrition. Relatedly, using next-of-kin interviews to determine mortality is subject to recall bias by the proxies. Second, we were limited to the seven chronic conditions assessed across all surveys. Therefore, we are likely overestimating the number of people without disease and underestimating the number of people with one disease and multimorbidity. Further, “respiratory problems” and “heart problems” could include multiple conditions, but were each counted as only one disease due to the structure of the questionnaires. Third, we did not allow for reversals in disease or disability status because the included diseases are all considered chronic. Participants were only asked if they had ever been diagnosed with a disease, and with panel data it is difficult to assess when and if reversals in disability occur. Lastly, the small sample size and number of transitions, particularly in the CRELES and MHAS data, resulted in wide confidence intervals, which precluded us from observing any clear patterns or finding statistically significant differences between several estimates. The small samples may also factor into our LE estimates being larger than those reported in vital statistics, but the lower bounds of our confidence intervals were fairly close to many vital statistics and estimates from other studies (Supplementary Material Section V).

In this study, we identified sex and educational inequalities in MMLE both within and between the countries of Costa Rica, Mexico, and the US. This approach allowed us to consider how macrolevel contextual determinants may be associated with microlevel health outcomes over time, and this should be further pursued in future research. The concept of MMLE, and the incorporation of disability status, can also be easily extended beyond what was done in this paper to include additional indicators of progression, such as using instrumental ADLs or cognitive function. MMLE is a valuable measure of population health and can be used to help better understand how the extent of multimorbidity inequalities and the aging process differs across contexts.

## Supplementary Material

Supplementary data are available at *The Journals of Gerontology, Series B: Psychological Sciences and Social Sciences* online.

gbae093_suppl_Supplementary_Materials

## Data Availability

This analysis uses data or information from the Harmonized CRELES (Costa Rican Study on Longevity and Healthy Aging) data set and Codebook, Version A as of August 2016, developed by the Gateway to Global Aging Data. CRELES data is available at http://creles.berkeley.edu/index.html. This analysis uses data or information from the Harmonized MHAS (Mexican Health and Aging Study) data set and Codebook, Version C as of September 2022, developed by the Gateway to Global Aging Data in collaboration with the MHAS research team. The Harmonized MHAS data files and documentation are for public use and available at www.MHASweb.org. Data files and documentation are public use and available at www.MHASweb.org. For more information about the Harmonization project, please refer to www.g2aging.org. This analysis uses data or information from the RAND HRS Longitudinal File 2018 (V1), which is an easy-to-use dataset based on the HRS core data. This file was developed and produced at the RAND Center for the Study of Aging. All HRS and RAND HRS data are available at https://hrsdata.isr.umich.edu/data-products/rand.
