# Supplementary material for: Inequalities in Disability-Free and Disabling Multimorbid Life Expectancy in Costa Rica, Mexico, and the United States
Source: J Gerontol B Psychol Sci Soc Sci. 2024 May 24;79(8):gbae093. doi: 10.1093/geronb/gbae093 (PMC11227002; doi:10.1093/geronb/gbae093)
Supplement: gbae093_suppl_Supplementary_Materials [file gbae093_suppl_supplementary_materials.docx]

**Supplementary Material**

**Table of Contents**

[Section I: Description of survey waves and reasons for exclusion 39](#_Toc167191864)

[Section II: Prevalence at origin state for ages 60-69 in Costa Rica, Mexico, and the United States 40](#_Toc167191865)

[Section III: Transition probability plots for female 41](#_Toc167191866)

[Section IV. Time spent in each destination state at age 60 from different origin states (health expectancy), total life expectancy from each origin state, and the health and life expectancy for each destination state averaged over all origin states 42](#_Toc167191867)

[Section V: Life expectancy comparison with vital statistics and other studies 54](#_Toc167191868)

[Section VI. Multimorbidity-free life expectancy, multimorbid life expectancy, and life expectancy at age 60 55](#_Toc167191869)

[Section VII: Evidence for cumulative (dis)advantage 56](#_Toc167191870)

[Section VIII: Sensitivity analysis excluding hypertension from multimorbidity definition 57](#_Toc167191871)

[References 58](#_Toc167191872)

# Section I: Description of survey waves and reasons for exclusion

**
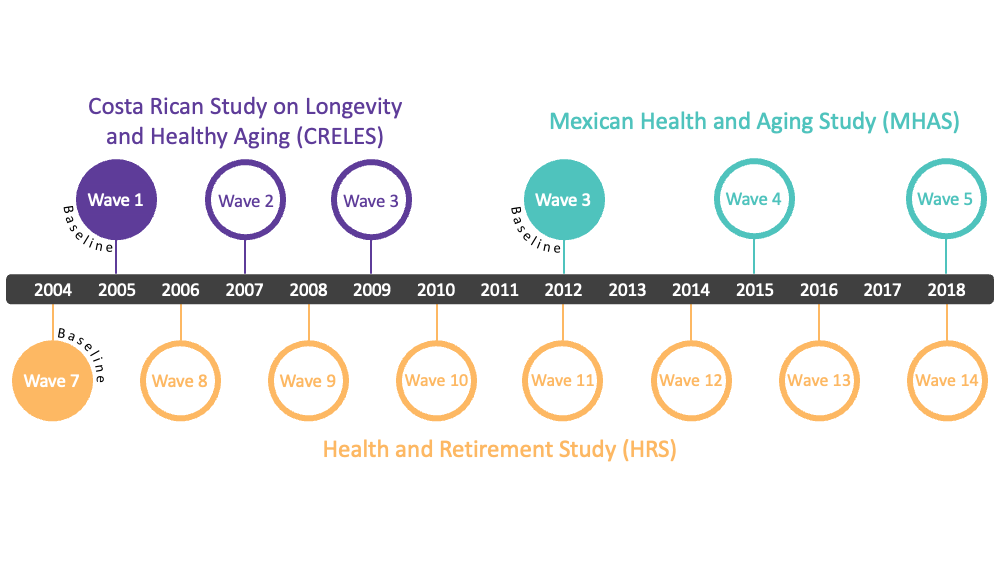
**

**
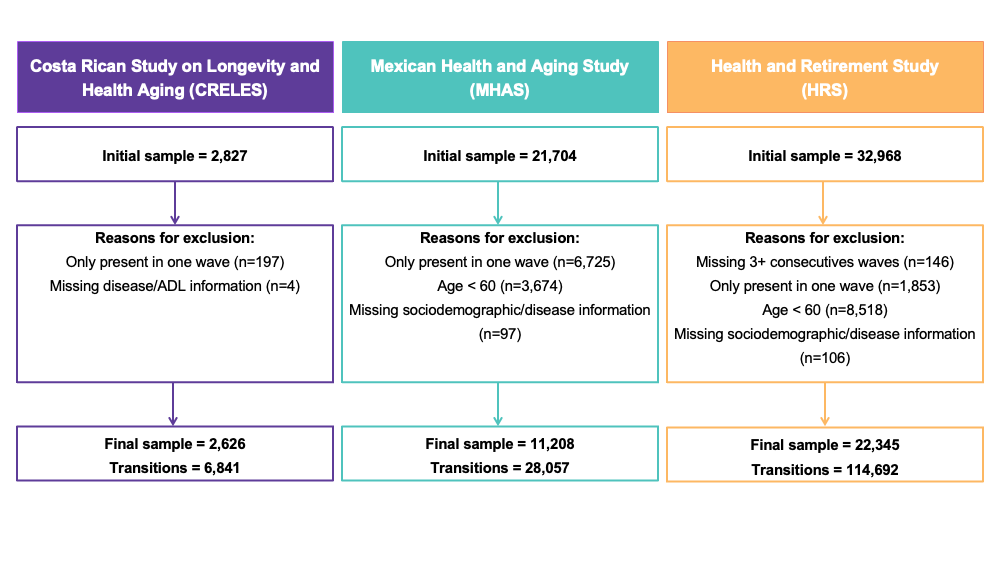
**

# Section II: Prevalence at origin state for ages 60-69 in Costa Rica, Mexico, and the United States


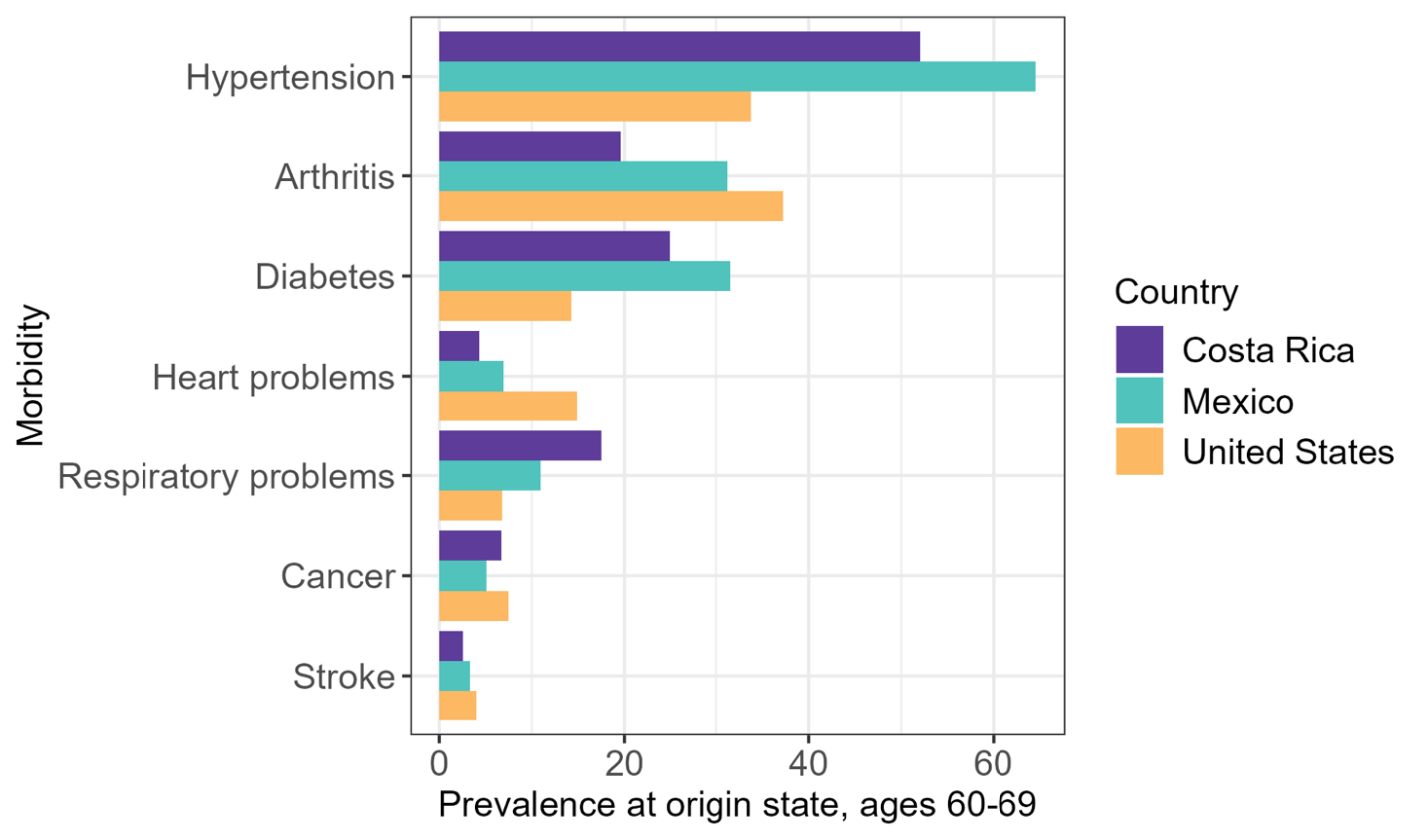


# Section III: Transition probability plots for female

**
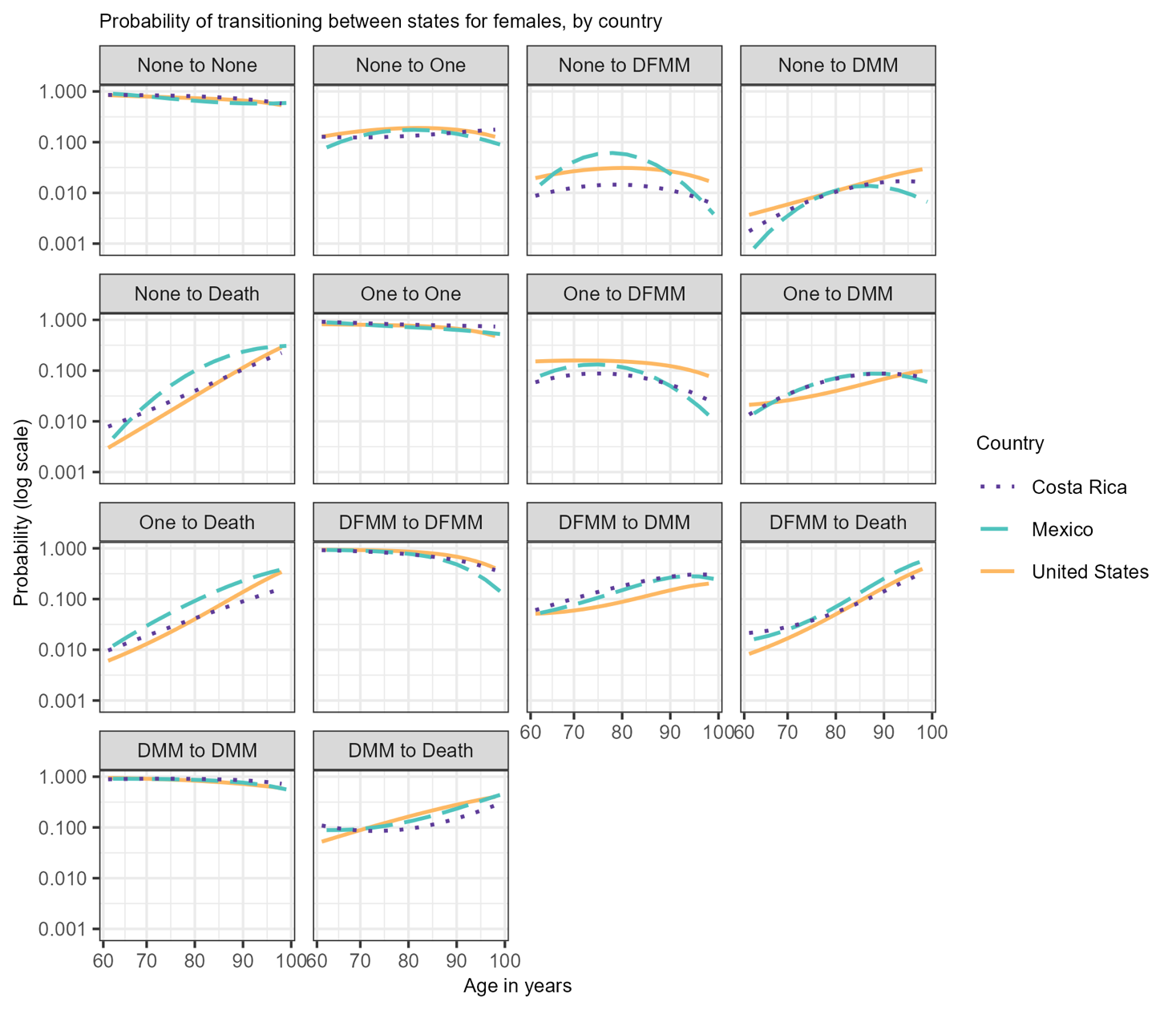
**

*Note.* None: no disease, One: one disease, DFMM: Disability-free multimorbidity, DMM: Disabling multimorbidity

# Section IV. Time spent in each destination state at age 60 from different origin states (health expectancy), total life expectancy from each origin state, and the health and life expectancy for each destination state averaged over all origin states

*Note.* Empty cells are present because those transitions are not possible in our analysis, e.g., the transition from 1 disease back to 0 disease.

## CRELES

**Male**

|  | **Origin state** | | | | | | | | | | | |  |  |  |
| --- | --- | --- | --- | --- | --- | --- | --- | --- | --- | --- | --- | --- | --- | --- | --- |
| **Destination state** | 0 disease | 95% CI | | 1 disease | 95% CI | | Disability-free Multimorbidity | 95% CI | | Disabling Multimorbidity | 95% CI | | **Average** | 95% CI | |
| 0 disease | 14.2 | 12.5 | 15.9 | 0.0 | 0.0 | 0.0 | 0.0 | 0.0 | 0.0 | 0.0 | 0.0 | 0.0 | 6.2 | 5.5 | 7.0 |
| 1 disease | 5.5 | 4.5 | 6.5 | 12.3 | 10.3 | 14.3 | 0.0 | 0.0 | 0.0 | 0.0 | 0.0 | 0.0 | 6.6 | 5.6 | 7.5 |
| Disability-free Multimorbidity | 3.4 | 2.7 | 4.0 | 6.2 | 5.1 | 7.2 | 13.0 | 10.8 | 15.2 | 0.0 | 0.0 | 0.0 | 6.0 | 5.1 | 6.9 |
| Disabling Multimorbidity | 3.5 | 2.4 | 4.5 | 5.9 | 4.5 | 7.3 | 8.2 | 5.9 | 10.5 | 13.5 | 8.2 | 18.7 | 5.5 | 4.2 | 6.9 |
| Total | 26.5 | 25.3 | 27.8 | 24.3 | 22.4 | 26.3 | 21.2 | 18.9 | 23.6 | 13.5 | 8.2 | 18.7 | 24.3 | 22.9 | 25.8 |

**Female**

|  | **Origin state** | | | | | | | | | | | |  |  |  |
| --- | --- | --- | --- | --- | --- | --- | --- | --- | --- | --- | --- | --- | --- | --- | --- |
| **Destination state** | 0 disease | 95% CI | | 1 disease | 95% CI | | Disability-free Multimorbidity | 95% CI | | Disabling Multimorbidity | 95% CI | | **Average** | 95% CI | |
| 0 disease | 10.9 | 8.5 | 13.3 | 0.0 | 0.0 | 0.0 | 0.0 | 0.0 | 0.0 | 0.0 | 0.0 | 0.0 | 2.5 | 1.9 | 3.0 |
| 1 disease | 8.2 | 6.8 | 9.5 | 13.2 | 11.4 | 15.0 | 0.0 | 0.0 | 0.0 | 0.0 | 0.0 | 0.0 | 7.5 | 6.6 | 8.5 |
| Disability-free Multimorbidity | 2.8 | 2.1 | 3.6 | 4.8 | 3.9 | 5.7 | 12.7 | 11.4 | 14.0 | 0.0 | 0.0 | 0.0 | 6.6 | 5.7 | 7.4 |
| Disabling Multimorbidity | 5.6 | 4.7 | 6.6 | 8.0 | 6.7 | 9.3 | 10.6 | 8.7 | 12.6 | 16.6 | 12.3 | 21.0 | 8.6 | 7.2 | 10.0 |
| Total | 27.5 | 26.0 | 29.0 | 26.0 | 24.1 | 27.8 | 23.3 | 20.9 | 25.7 | 16.6 | 12.3 | 21.0 | 25.1 | 23.3 | 26.9 |

**Male - Primary education**

|  | **Origin state** | | | | | | | | | | | |  |  |  |
| --- | --- | --- | --- | --- | --- | --- | --- | --- | --- | --- | --- | --- | --- | --- | --- |
| **Destination state** | 0 disease | 95% CI | | 1 disease | 95% CI | | Disability-free Multimorbidity | 95% CI | | Disabling Multimorbidity | 95% CI | | **Average** | 95% CI | |
| 0 disease | 13.3 | 11.5 | 15.1 | 0.0 | 0.0 | 0.0 | 0.0 | 0.0 | 0.0 | 0.0 | 0.0 | 0.0 | 5.8 | 5.0 | 6.6 |
| 1 disease | 5.9 | 4.8 | 6.9 | 12.4 | 10.3 | 14.5 | 0.0 | 0.0 | 0.0 | 0.0 | 0.0 | 0.0 | 6.7 | 5.7 | 7.8 |
| Disability-free Multimorbidity | 3.3 | 2.6 | 3.9 | 5.6 | 4.8 | 6.5 | 12.1 | 9.8 | 14.4 | 0.0 | 0.0 | 0.0 | 5.7 | 4.8 | 6.5 |
| Disabling Multimorbidity | 3.7 | 2.7 | 4.8 | 6.1 | 4.7 | 7.6 | 8.7 | 6.2 | 11.2 | 13.8 | 8.5 | 19.0 | 5.8 | 4.4 | 7.3 |
| Total | 26.1 | 24.8 | 27.5 | 24.2 | 22.2 | 26.1 | 20.8 | 18.2 | 23.4 | 13.8 | 8.5 | 19.0 | 24.0 | 22.4 | 25.7 |

**Male - Secondary education**

|  | **Origin state** | | | | | | | | | | | |  |  |  |
| --- | --- | --- | --- | --- | --- | --- | --- | --- | --- | --- | --- | --- | --- | --- | --- |
| **Destination state** | 0 disease | 95% CI | | 1 disease | 95% CI | | Disability-free Multimorbidity | 95% CI | | Disabling Multimorbidity | 95% CI | | **Average** | 95% CI | |
| 0 disease | 20.5 | 12.7 | 28.3 | 0.0 | 0.0 | 0.0 | 0.0 | 0.0 | 0.0 | 0.0 | 0.0 | 0.0 | 9.0 | 5.6 | 12.4 |
| 1 disease | 4.9 | 2.0 | 7.8 | 14.6 | 10.2 | 19.0 | 0.0 | 0.0 | 0.0 | 0.0 | 0.0 | 0.0 | 7.1 | 4.6 | 9.5 |
| Disability-free Multimorbidity | 2.1 | 0.3 | 3.8 | 6.0 | 2.7 | 9.4 | 14.7 | 8.8 | 20.6 | 0.0 | 0.0 | 0.0 | 5.8 | 3.0 | 8.5 |
| Disabling Multimorbidity | 0.9 | 0.0 | 2.3 | 2.1 | 0.0 | 5.3 | 3.4 | 0.0 | 8.2 | 6.7 | 0.0 | 15.1 | 2.0 | 0.0 | 4.8 |
| Total | 28.4 | 22.6 | 34.1 | 22.8 | 17.0 | 28.5 | 18.1 | 12.6 | 23.5 | 6.7 | 0.0 | 15.1 | 23.8 | 18.8 | 28.8 |

**Male - Post-secondary education**

|  | **Origin state** | | | | | | | | | | | |  |  |  |
| --- | --- | --- | --- | --- | --- | --- | --- | --- | --- | --- | --- | --- | --- | --- | --- |
| **Destination state** | 0 disease | 95% CI | | 1 disease | 95% CI | | Disability-free Multimorbidity | 95% CI | | Disabling Multimorbidity | 95% CI | | **Average** | 95% CI | |
| 0 disease | 16.3 | 8.1 | 24.4 | 0.0 | 0.0 | 0.0 | 0.0 | 0.0 | 0.0 | 0.0 | 0.0 | 0.0 | 7.1 | 3.6 | 10.7 |
| 1 disease | 3.3 | 1.2 | 5.5 | 8.6 | 5.3 | 11.8 | 0.0 | 0.0 | 0.0 | 0.0 | 0.0 | 0.0 | 4.3 | 2.4 | 6.2 |
| Disability-free Multimorbidity | 5.8 | 2.9 | 8.8 | 11.1 | 4.9 | 17.3 | 17.4 | 10.2 | 24.7 | 0.0 | 0.0 | 0.0 | 9.6 | 5.3 | 14.0 |
| Disabling Multimorbidity | 4.5 | 0.0 | 9.2 | 8.2 | 1.8 | 14.7 | 9.8 | 2.4 | 17.2 | 18.2 | 7.4 | 29.0 | 7.2 | 1.4 | 13.1 |
| Total | 30.0 | 24.4 | 35.5 | 27.9 | 22.1 | 33.7 | 27.2 | 20.6 | 33.8 | 18.2 | 7.4 | 29.0 | 28.4 | 22.7 | 34.0 |

**Female - Primary education**

|  | **Origin state** | | | | | | | | | | | |  |  |  |
| --- | --- | --- | --- | --- | --- | --- | --- | --- | --- | --- | --- | --- | --- | --- | --- |
| **Destination state** | 0 disease | 95% CI | | 1 disease | 95% CI | | Disability-free Multimorbidity | 95% CI | | Disabling Multimorbidity | 95% CI | | **Average** | 95% CI | |
| 0 disease | 11.0 | 8.5 | 13.5 | 0.0 | 0.0 | 0.0 | 0.0 | 0.0 | 0.0 | 0.0 | 0.0 | 0.0 | 2.5 | 2.0 | 3.1 |
| 1 disease | 7.9 | 6.6 | 9.1 | 12.9 | 11.2 | 14.6 | 0.0 | 0.0 | 0.0 | 0.0 | 0.0 | 0.0 | 7.3 | 6.4 | 8.2 |
| Disability-free Multimorbidity | 2.6 | 1.9 | 3.3 | 4.4 | 3.6 | 5.3 | 11.8 | 10.4 | 13.2 | 0.0 | 0.0 | 0.0 | 6.1 | 5.2 | 6.9 |
| Disabling Multimorbidity | 6.1 | 4.8 | 7.4 | 8.8 | 7.2 | 10.4 | 11.8 | 9.6 | 13.9 | 18.0 | 13.6 | 22.4 | 9.5 | 7.8 | 11.1 |
| Total | 27.6 | 25.9 | 29.3 | 26.2 | 24.3 | 28.0 | 23.6 | 20.9 | 26.2 | 18.0 | 13.6 | 22.4 | 25.4 | 23.4 | 27.3 |

**Female - Secondary education**

|  | **Origin state** | | | | | | | | | | | |  |  |  |
| --- | --- | --- | --- | --- | --- | --- | --- | --- | --- | --- | --- | --- | --- | --- | --- |
| **Destination state** | 0 disease | 95% CI | | 1 disease | 95% CI | | Disability-free Multimorbidity | 95% CI | | Disabling Multimorbidity | 95% CI | | **Average** | 95% CI | |
| 0 disease | 11.3 | 4.0 | 18.5 | 0.0 | 0.0 | 0.0 | 0.0 | 0.0 | 0.0 | 0.0 | 0.0 | 0.0 | 2.6 | 0.9 | 4.2 |
| 1 disease | 7.1 | 3.6 | 10.7 | 11.7 | 6.8 | 16.6 | 0.0 | 0.0 | 0.0 | 0.0 | 0.0 | 0.0 | 6.7 | 3.9 | 9.4 |
| Disability-free Multimorbidity | 4.9 | 0.9 | 9.0 | 8.4 | 3.3 | 13.6 | 17.4 | 12.3 | 22.4 | 0.0 | 0.0 | 0.0 | 10.0 | 5.5 | 14.5 |
| Disabling Multimorbidity | 3.3 | 1.0 | 5.5 | 4.7 | 1.3 | 8.2 | 5.9 | 1.5 | 10.3 | 11.7 | 3.6 | 19.8 | 5.0 | 1.5 | 8.6 |
| Total | 26.6 | 21.7 | 31.5 | 24.9 | 20.2 | 29.6 | 23.3 | 17.3 | 29.2 | 11.7 | 3.6 | 19.8 | 24.3 | 19.4 | 29.2 |

**Female - Post-secondary education**

|  | **Origin state** | | | | | | | | | | | |  |  |  |
| --- | --- | --- | --- | --- | --- | --- | --- | --- | --- | --- | --- | --- | --- | --- | --- |
| **Destination state** | 0 disease | 95% CI | | 1 disease | 95% CI | | Disability-free Multimorbidity | 95% CI | | Disabling Multimorbidity | 95% CI | | **Average** | 95% CI | |
| 0 disease | 8.7 | 2.5 | 14.9 | 0.0 | 0.0 | 0.0 | 0.0 | 0.0 | 0.0 | 0.0 | 0.0 | 0.0 | 2.0 | 0.6 | 3.4 |
| 1 disease | 14.9 | 8.1 | 21.7 | 20.4 | 12.2 | 28.6 | 0.0 | 0.0 | 0.0 | 0.0 | 0.0 | 0.0 | 12.2 | 7.2 | 17.1 |
| Disability-free Multimorbidity | 3.4 | 0.2 | 6.6 | 5.0 | 1.1 | 8.9 | 16.9 | 11.5 | 22.3 | 0.0 | 0.0 | 0.0 | 8.0 | 4.3 | 11.8 |
| Disabling Multimorbidity | 2.2 | 0.0 | 4.8 | 2.8 | 0.0 | 6.1 | 5.0 | 0.0 | 10.3 | 10.0 | 0.2 | 19.7 | 3.6 | 0.0 | 7.5 |
| Total | 29.2 | 22.8 | 35.6 | 28.2 | 21.6 | 34.8 | 21.9 | 14.3 | 29.5 | 10.0 | 0.2 | 19.7 | 25.8 | 19.5 | 32.1 |

## MHAS

**Male**

|  | **Origin state** | | | | | | | | | | | |  |  |  |
| --- | --- | --- | --- | --- | --- | --- | --- | --- | --- | --- | --- | --- | --- | --- | --- |
| **Destination state** | 0 disease | 95% CI | | 1 disease | 95% CI | | Disability-free Multimorbidity | 95% CI | | Disabling Multimorbidity | 95% CI | | **Average** | 95% CI | |
| 0 disease | 12.4 | 11.0 | 13.8 | 0.0 | 0.0 | 0.0 | 0.0 | 0.0 | 0.0 | 0.0 | 0.0 | 0.0 | 4.9 | 4.4 | 5.5 |
| 1 disease | 6.5 | 5.4 | 7.5 | 12.7 | 11.2 | 14.2 | 0.0 | 0.0 | 0.0 | 0.0 | 0.0 | 0.0 | 6.6 | 5.8 | 7.3 |
| Disability-free Multimorbidity | 3.3 | 2.6 | 4.0 | 6.4 | 5.3 | 7.4 | 15.2 | 13.9 | 16.6 | 0.0 | 0.0 | 0.0 | 7.0 | 6.2 | 7.7 |
| Disabling Multimorbidity | 2.6 | 1.9 | 3.2 | 4.0 | 3.2 | 4.8 | 5.7 | 4.4 | 6.9 | 16.2 | 13.2 | 19.2 | 4.4 | 3.6 | 5.3 |
| Total | 24.8 | 23.7 | 25.8 | 23.1 | 21.8 | 24.3 | 20.9 | 19.1 | 22.7 | 16.2 | 13.2 | 19.2 | 22.9 | 21.8 | 24.0 |

**Female**

|  | **Origin state** | | | | | | | | | | | |  |  |  |
| --- | --- | --- | --- | --- | --- | --- | --- | --- | --- | --- | --- | --- | --- | --- | --- |
| **Destination state** | 0 disease | 95% CI | | 1 disease | 95% CI | | Disability-free Multimorbidity | 95% CI | | Disabling Multimorbidity | 95% CI | | **Average** | 95% CI | |
| 0 disease | 12.8 | 11.4 | 14.1 | 0.0 | 0.0 | 0.0 | 0.0 | 0.0 | 0.0 | 0.0 | 0.0 | 0.0 | 2.5 | 2.3 | 2.8 |
| 1 disease | 6.2 | 5.2 | 7.3 | 13.6 | 12.5 | 14.6 | 0.0 | 0.0 | 0.0 | 0.0 | 0.0 | 0.0 | 5.6 | 5.2 | 6.1 |
| Disability-free Multimorbidity | 3.8 | 3.0 | 4.6 | 6.3 | 5.4 | 7.1 | 17.0 | 15.9 | 18.1 | 0.0 | 0.0 | 0.0 | 9.3 | 8.6 | 9.9 |
| Disabling Multimorbidity | 3.8 | 3.1 | 4.6 | 6.1 | 5.2 | 7.0 | 8.7 | 7.5 | 9.8 | 19.6 | 17.4 | 21.8 | 7.9 | 7.0 | 8.9 |
| Total | 26.6 | 25.4 | 27.9 | 25.9 | 24.9 | 26.9 | 25.7 | 24.5 | 26.8 | 19.6 | 17.4 | 21.8 | 25.4 | 24.4 | 26.3 |

**Male - Primary education**

|  | **Origin state** | | | | | | | | | | | |  |  |  |
| --- | --- | --- | --- | --- | --- | --- | --- | --- | --- | --- | --- | --- | --- | --- | --- |
| **Destination state** | 0 disease | 95% CI | | 1 disease | 95% CI | | Disability-free Multimorbidity | 95% CI | | Disabling Multimorbidity | 95% CI | | **Average** | 95% CI | |
| 0 disease | 13.1 | 11.3 | 14.8 | 0.0 | 0.0 | 0.0 | 0.0 | 0.0 | 0.0 | 0.0 | 0.0 | 0.0 | 5.1 | 4.4 | 5.7 |
| 1 disease | 6.3 | 5.0 | 7.5 | 12.4 | 10.8 | 14.0 | 0.0 | 0.0 | 0.0 | 0.0 | 0.0 | 0.0 | 6.4 | 5.6 | 7.3 |
| Disability-free Multimorbidity | 3.0 | 2.4 | 3.7 | 6.1 | 5.0 | 7.2 | 14.5 | 13.2 | 15.9 | 0.0 | 0.0 | 0.0 | 6.7 | 5.9 | 7.4 |
| Disabling Multimorbidity | 2.6 | 1.9 | 3.3 | 4.2 | 3.2 | 5.1 | 5.9 | 4.5 | 7.2 | 16.3 | 13.1 | 19.5 | 4.6 | 3.7 | 5.5 |
| Total | 24.9 | 23.8 | 26.1 | 22.7 | 21.3 | 24.0 | 20.4 | 18.7 | 22.1 | 16.3 | 13.1 | 19.5 | 22.7 | 21.5 | 23.8 |

**Male - Secondary education**

|  | **Origin state** | | | | | | | | | | | |  |  |  |
| --- | --- | --- | --- | --- | --- | --- | --- | --- | --- | --- | --- | --- | --- | --- | --- |
| **Destination state** | 0 disease | 95% CI | | 1 disease | 95% CI | | Disability-free Multimorbidity | 95% CI | | Disabling Multimorbidity | 95% CI | | **Average** | 95% CI | |
| 0 disease | 12.4 | 9.1 | 15.7 | 0.0 | 0.0 | 0.0 | 0.0 | 0.0 | 0.0 | 0.0 | 0.0 | 0.0 | 4.9 | 3.6 | 6.2 |
| 1 disease | 8.3 | 5.7 | 10.9 | 15.0 | 11.6 | 18.4 | 0.0 | 0.0 | 0.0 | 0.0 | 0.0 | 0.0 | 8.0 | 6.0 | 10.0 |
| Disability-free Multimorbidity | 3.5 | 1.2 | 5.9 | 6.5 | 3.1 | 9.8 | 16.6 | 12.0 | 21.2 | 0.0 | 0.0 | 0.0 | 7.4 | 4.4 | 10.4 |
| Disabling Multimorbidity | 2.4 | 0.6 | 4.1 | 3.6 | 1.2 | 6.0 | 5.5 | 2.0 | 8.9 | 16.6 | 9.4 | 23.7 | 4.2 | 1.6 | 6.7 |
| Total | 26.6 | 22.9 | 30.3 | 25.0 | 20.8 | 29.2 | 22.0 | 15.8 | 28.3 | 16.6 | 9.4 | 23.7 | 24.5 | 20.1 | 28.9 |

**Male - Post-secondary education**

|  | **Origin state** | | | | | | | | | | | |  |  |  |
| --- | --- | --- | --- | --- | --- | --- | --- | --- | --- | --- | --- | --- | --- | --- | --- |
| **Destination state** | 0 disease | 95% CI | | 1 disease | 95% CI | | Disability-free Multimorbidity | 95% CI | | Disabling Multimorbidity | 95% CI | | **Average** | 95% CI | |
| 0 disease | 9.5 | 5.8 | 13.1 | 0.0 | 0.0 | 0.0 | 0.0 | 0.0 | 0.0 | 0.0 | 0.0 | 0.0 | 3.7 | 2.3 | 5.2 |
| 1 disease | 6.1 | 2.5 | 9.7 | 10.9 | 5.8 | 16.0 | 0.0 | 0.0 | 0.0 | 0.0 | 0.0 | 0.0 | 5.9 | 2.9 | 8.8 |
| Disability-free Multimorbidity | 5.7 | 1.6 | 9.9 | 9.3 | 3.5 | 15.1 | 18.4 | 13.0 | 23.7 | 0.0 | 0.0 | 0.0 | 9.6 | 5.0 | 14.2 |
| Disabling Multimorbidity | 1.5 | 0.2 | 2.7 | 2.0 | 0.4 | 3.7 | 2.6 | 0.5 | 4.7 | 11.0 | 4.9 | 17.1 | 2.4 | 0.6 | 4.2 |
| Total | 22.8 | 18.1 | 27.5 | 22.2 | 17.7 | 26.7 | 21.0 | 15.1 | 26.9 | 11.0 | 4.9 | 17.1 | 21.6 | 16.9 | 26.2 |

**Female - Primary education**

|  | **Origin state** | | | | | | | | | | | |  |  |  |
| --- | --- | --- | --- | --- | --- | --- | --- | --- | --- | --- | --- | --- | --- | --- | --- |
| **Destination state** | 0 disease | 95% CI | | 1 disease | 95% CI | | Disability-free Multimorbidity | 95% CI | | Disabling Multimorbidity | 95% CI | | **Average** | 95% CI | |
| 0 disease | 12.4 | 10.9 | 13.8 | 0.0 | 0.0 | 0.0 | 0.0 | 0.0 | 0.0 | 0.0 | 0.0 | 0.0 | 2.4 | 2.2 | 2.7 |
| 1 disease | 6.2 | 5.2 | 7.3 | 13.4 | 12.3 | 14.6 | 0.0 | 0.0 | 0.0 | 0.0 | 0.0 | 0.0 | 5.6 | 5.1 | 6.1 |
| Disability-free Multimorbidity | 3.8 | 3.0 | 4.7 | 6.2 | 5.3 | 7.1 | 16.8 | 15.8 | 17.8 | 0.0 | 0.0 | 0.0 | 9.2 | 8.5 | 9.9 |
| Disabling Multimorbidity | 3.9 | 3.1 | 4.7 | 6.2 | 5.2 | 7.1 | 8.8 | 7.6 | 9.9 | 19.6 | 17.4 | 21.9 | 8.0 | 7.1 | 9.0 |
| Total | 26.4 | 25.1 | 27.7 | 25.8 | 24.8 | 26.8 | 25.6 | 24.4 | 26.7 | 19.6 | 17.4 | 21.9 | 25.2 | 24.2 | 26.2 |

**Female - Secondary education**

|  | **Origin state** | | | | | | | | | | | |  |  |  |
| --- | --- | --- | --- | --- | --- | --- | --- | --- | --- | --- | --- | --- | --- | --- | --- |
| **Destination state** | 0 disease | 95% CI | | 1 disease | 95% CI | | Disability-free Multimorbidity | 95% CI | | Disabling Multimorbidity | 95% CI | | **Average** | 95% CI | |
| 0 disease | 15.5 | 11.4 | 19.6 | 0.0 | 0.0 | 0.0 | 0.0 | 0.0 | 0.0 | 0.0 | 0.0 | 0.0 | 3.1 | 2.2 | 3.9 |
| 1 disease | 5.7 | 3.4 | 8.0 | 13.8 | 10.9 | 16.7 | 0.0 | 0.0 | 0.0 | 0.0 | 0.0 | 0.0 | 5.6 | 4.3 | 6.9 |
| Disability-free Multimorbidity | 3.6 | 1.8 | 5.3 | 6.9 | 4.1 | 9.6 | 18.0 | 14.4 | 21.6 | 0.0 | 0.0 | 0.0 | 9.8 | 7.4 | 12.3 |
| Disabling Multimorbidity | 3.2 | 1.4 | 5.0 | 5.8 | 2.9 | 8.7 | 8.2 | 4.1 | 12.2 | 19.5 | 14.1 | 24.9 | 7.5 | 4.3 | 10.7 |
| Total | 28.0 | 25.3 | 30.7 | 26.5 | 23.7 | 29.2 | 26.2 | 23.2 | 29.1 | 19.5 | 14.1 | 24.9 | 26.0 | 23.2 | 28.8 |

**Female - Post-secondary education**

|  | **Origin state** | | | | | | | | | | | |  |  |  |
| --- | --- | --- | --- | --- | --- | --- | --- | --- | --- | --- | --- | --- | --- | --- | --- |
| **Destination state** | 0 disease | 95% CI | | 1 disease | 95% CI | | Disability-free Multimorbidity | 95% CI | | Disabling Multimorbidity | 95% CI | | **Average** | 95% CI | |
| 0 disease | 10.2 | 5.2 | 15.2 | 0.0 | 0.0 | 0.0 | 0.0 | 0.0 | 0.0 | 0.0 | 0.0 | 0.0 | 2.0 | 1.0 | 3.0 |
| 1 disease | 9.8 | 3.8 | 15.8 | 16.8 | 10.5 | 23.2 | 0.0 | 0.0 | 0.0 | 0.0 | 0.0 | 0.0 | 7.4 | 4.2 | 10.6 |
| Disability-free Multimorbidity | 4.5 | 1.2 | 7.8 | 6.1 | 1.7 | 10.5 | 18.6 | 13.2 | 24.0 | 0.0 | 0.0 | 0.0 | 10.0 | 6.1 | 13.9 |
| Disabling Multimorbidity | 3.9 | 0.4 | 7.4 | 5.2 | 0.8 | 9.7 | 8.3 | 2.4 | 14.2 | 20.1 | 10.2 | 30.0 | 7.6 | 2.4 | 12.7 |
| Total | 28.4 | 22.4 | 34.4 | 28.2 | 22.1 | 34.3 | 26.9 | 20.0 | 33.8 | 20.1 | 10.2 | 30.0 | 26.9 | 20.4 | 33.4 |

## HRS

**Male**

|  | **Origin state** | | | | | | | | | | | |  |  |  |
| --- | --- | --- | --- | --- | --- | --- | --- | --- | --- | --- | --- | --- | --- | --- | --- |
| **Destination state** | 0 disease | 95% CI | | 1 disease | 95% CI | | Disability-free Multimorbidity | 95% CI | | Disabling Multimorbidity | 95% CI | | **Average** | 95% CI | |
| 0 disease | 8.1 | 7.6 | 8.6 | 0.0 | 0.0 | 0.0 | 0.0 | 0.0 | 0.0 | 0.0 | 0.0 | 0.0 | 1.4 | 1.3 | 1.5 |
| 1 disease | 5.6 | 5.3 | 5.9 | 8.3 | 7.9 | 8.7 | 0.0 | 0.0 | 0.0 | 0.0 | 0.0 | 0.0 | 3.2 | 3.0 | 3.3 |
| Disability-free Multimorbidity | 7.3 | 7.0 | 7.7 | 10.7 | 10.4 | 11.1 | 17.3 | 16.9 | 17.8 | 0.0 | 0.0 | 0.0 | 11.9 | 11.6 | 12.2 |
| Disabling Multimorbidity | 2.3 | 2.2 | 2.5 | 3.1 | 2.9 | 3.3 | 3.7 | 3.5 | 4.0 | 13.0 | 12.3 | 13.6 | 4.4 | 4.1 | 4.6 |
| Total | 23.4 | 23.0 | 23.7 | 22.1 | 21.8 | 22.5 | 21.1 | 20.7 | 21.5 | 13.0 | 12.3 | 13.6 | 20.8 | 20.5 | 21.2 |

**Female**

|  | **Origin state** | | | | | | | | | | | |  |  |  |
| --- | --- | --- | --- | --- | --- | --- | --- | --- | --- | --- | --- | --- | --- | --- | --- |
| **Destination state** | 0 disease | 95% CI | | 1 disease | 95% CI | | Disability-free Multimorbidity | 95% CI | | Disabling Multimorbidity | 95% CI | | **Average** | 95% CI | |
| 0 disease | 9.1 | 8.6 | 9.7 | 0.0 | 0.0 | 0.0 | 0.0 | 0.0 | 0.0 | 0.0 | 0.0 | 0.0 | 1.3 | 1.2 | 1.4 |
| 1 disease | 6.8 | 6.4 | 7.1 | 9.0 | 8.6 | 9.4 | 0.0 | 0.0 | 0.0 | 0.0 | 0.0 | 0.0 | 3.3 | 3.1 | 3.4 |
| Disability-free Multimorbidity | 6.8 | 6.5 | 7.2 | 10.4 | 10.1 | 10.8 | 16.9 | 16.5 | 17.3 | 0.0 | 0.0 | 0.0 | 11.1 | 10.9 | 11.4 |
| Disabling Multimorbidity | 3.9 | 3.7 | 4.1 | 5.4 | 5.1 | 5.6 | 6.6 | 6.3 | 6.9 | 16.2 | 15.6 | 16.8 | 7.4 | 7.2 | 7.7 |
| Total | 26.6 | 26.2 | 26.9 | 24.8 | 24.5 | 25.1 | 23.5 | 23.2 | 23.9 | 16.2 | 15.6 | 16.8 | 23.1 | 22.8 | 23.4 |

**Male - Primary education**

|  | **Origin state** | | | | | | | | | | | |  |  |  |
| --- | --- | --- | --- | --- | --- | --- | --- | --- | --- | --- | --- | --- | --- | --- | --- |
| **Destination state** | 0 disease | 95% CI | | 1 disease | 95% CI | | Disability-free Multimorbidity | 95% CI | | Disabling Multimorbidity | 95% CI | | **Average** | 95% CI | |
| 0 disease | 8.6 | 6.5 | 10.6 | 0.0 | 0.0 | 0.0 | 0.0 | 0.0 | 0.0 | 0.0 | 0.0 | 0.0 | 1.5 | 1.1 | 1.9 |
| 1 disease | 4.9 | 3.9 | 6.0 | 7.7 | 6.5 | 9.0 | 0.0 | 0.0 | 0.0 | 0.0 | 0.0 | 0.0 | 2.9 | 2.4 | 3.4 |
| Disability-free Multimorbidity | 5.6 | 4.6 | 6.6 | 8.5 | 7.3 | 9.7 | 14.2 | 12.9 | 15.4 | 0.0 | 0.0 | 0.0 | 9.6 | 8.6 | 10.5 |
| Disabling Multimorbidity | 3.6 | 2.7 | 4.5 | 5.0 | 4.0 | 6.0 | 6.0 | 4.9 | 7.2 | 15.3 | 13.7 | 16.9 | 6.4 | 5.3 | 7.4 |
| Total | 22.7 | 21.4 | 23.9 | 21.2 | 20.1 | 22.3 | 20.2 | 19.0 | 21.4 | 15.3 | 13.7 | 16.9 | 20.3 | 19.3 | 21.4 |

**Male - Secondary education**

|  | **Origin state** | | | | | | | | | | | |  |  |  |
| --- | --- | --- | --- | --- | --- | --- | --- | --- | --- | --- | --- | --- | --- | --- | --- |
| **Destination state** | 0 disease | 95% CI | | 1 disease | 95% CI | | Disability-free Multimorbidity | 95% CI | | Disabling Multimorbidity | 95% CI | | **Average** | 95% CI | |
| 0 disease | 7.5 | 6.8 | 8.3 | 0.0 | 0.0 | 0.0 | 0.0 | 0.0 | 0.0 | 0.0 | 0.0 | 0.0 | 1.3 | 1.2 | 1.5 |
| 1 disease | 5.4 | 4.9 | 5.8 | 8.0 | 7.5 | 8.5 | 0.0 | 0.0 | 0.0 | 0.0 | 0.0 | 0.0 | 3.1 | 2.9 | 3.3 |
| Disability-free Multimorbidity | 6.8 | 6.3 | 7.3 | 9.8 | 9.3 | 10.3 | 16.0 | 15.5 | 16.6 | 0.0 | 0.0 | 0.0 | 10.9 | 10.5 | 11.3 |
| Disabling Multimorbidity | 2.4 | 2.1 | 2.6 | 3.1 | 2.9 | 3.4 | 3.8 | 3.5 | 4.1 | 12.5 | 11.8 | 13.2 | 4.4 | 4.1 | 4.7 |
| Total | 22.1 | 21.5 | 22.6 | 20.9 | 20.5 | 21.4 | 19.9 | 19.4 | 20.4 | 12.5 | 11.8 | 13.2 | 19.7 | 19.3 | 20.1 |

**Male - Post-secondary education**

|  | **Origin state** | | | | | | | | | | | |  |  |  |
| --- | --- | --- | --- | --- | --- | --- | --- | --- | --- | --- | --- | --- | --- | --- | --- |
| **Destination state** | 0 disease | 95% CI | | 1 disease | 95% CI | | Disability-free Multimorbidity | 95% CI | | Disabling Multimorbidity | 95% CI | | **Average** | 95% CI | |
| 0 disease | 8.4 | 7.7 | 9.1 | 0.0 | 0.0 | 0.0 | 0.0 | 0.0 | 0.0 | 0.0 | 0.0 | 0.0 | 1.5 | 1.4 | 1.6 |
| 1 disease | 5.9 | 5.4 | 6.4 | 8.5 | 8.0 | 9.1 | 0.0 | 0.0 | 0.0 | 0.0 | 0.0 | 0.0 | 3.3 | 3.1 | 3.5 |
| Disability-free Multimorbidity | 8.3 | 7.7 | 8.8 | 12.1 | 11.5 | 12.7 | 19.0 | 18.4 | 19.6 | 0.0 | 0.0 | 0.0 | 13.1 | 12.7 | 13.6 |
| Disabling Multimorbidity | 2.2 | 1.9 | 2.4 | 2.9 | 2.6 | 3.1 | 3.5 | 3.1 | 3.8 | 13.1 | 12.3 | 13.9 | 4.2 | 3.8 | 4.5 |
| Total | 24.8 | 24.2 | 25.3 | 23.5 | 23.0 | 24.0 | 22.5 | 21.9 | 23.0 | 13.1 | 12.3 | 13.9 | 22.1 | 21.6 | 22.6 |

**Female - Primary education**

|  | **Origin state** | | | | | | | | | | | |  |  |  |
| --- | --- | --- | --- | --- | --- | --- | --- | --- | --- | --- | --- | --- | --- | --- | --- |
| **Destination state** | 0 disease | 95% CI | | 1 disease | 95% CI | | Disability-free Multimorbidity | 95% CI | | Disabling Multimorbidity | 95% CI | | **Average** | 95% CI | |
| 0 disease | 5.3 | 3.6 | 7.0 | 0.0 | 0.0 | 0.0 | 0.0 | 0.0 | 0.0 | 0.0 | 0.0 | 0.0 | 0.8 | 0.5 | 1.0 |
| 1 disease | 6.0 | 4.6 | 7.3 | 7.5 | 6.0 | 8.9 | 0.0 | 0.0 | 0.0 | 0.0 | 0.0 | 0.0 | 2.8 | 2.2 | 3.3 |
| Disability-free Multimorbidity | 5.7 | 4.5 | 6.9 | 7.3 | 6.0 | 8.5 | 12.2 | 10.8 | 13.5 | 0.0 | 0.0 | 0.0 | 8.1 | 7.0 | 9.1 |
| Disabling Multimorbidity | 7.8 | 6.6 | 9.0 | 9.4 | 8.2 | 10.5 | 11.1 | 9.8 | 12.4 | 19.7 | 18.4 | 21.1 | 11.6 | 10.4 | 12.8 |
| Total | 24.8 | 23.5 | 26.0 | 24.1 | 23.0 | 25.3 | 23.3 | 22.1 | 24.5 | 19.7 | 18.4 | 21.1 | 23.2 | 22.0 | 24.3 |

**Female - Secondary education**

|  | **Origin state** | | | | | | | | | | | |  |  |  |
| --- | --- | --- | --- | --- | --- | --- | --- | --- | --- | --- | --- | --- | --- | --- | --- |
| **Destination state** | 0 disease | 95% CI | | 1 disease | 95% CI | | Disability-free Multimorbidity | 95% CI | | Disabling Multimorbidity | 95% CI | | **Average** | 95% CI | |
| 0 disease | 8.6 | 7.8 | 9.3 | 0.0 | 0.0 | 0.0 | 0.0 | 0.0 | 0.0 | 0.0 | 0.0 | 0.0 | 1.2 | 1.1 | 1.3 |
| 1 disease | 6.7 | 6.2 | 7.1 | 8.8 | 8.3 | 9.4 | 0.0 | 0.0 | 0.0 | 0.0 | 0.0 | 0.0 | 3.2 | 3.0 | 3.4 |
| Disability-free Multimorbidity | 6.6 | 6.1 | 7.0 | 9.8 | 9.4 | 10.2 | 16.1 | 15.6 | 16.6 | 0.0 | 0.0 | 0.0 | 10.6 | 10.2 | 10.9 |
| Disabling Multimorbidity | 3.9 | 3.6 | 4.2 | 5.3 | 5.0 | 5.6 | 6.6 | 6.2 | 6.9 | 15.7 | 15.1 | 16.3 | 7.3 | 7.0 | 7.7 |
| Total | 25.7 | 25.3 | 26.2 | 24.0 | 23.6 | 24.4 | 22.7 | 22.3 | 23.1 | 15.7 | 15.1 | 16.3 | 22.3 | 22.0 | 22.7 |

**Female - Post-secondary education**

|  | **Origin state** | | | | | | | | | | | |  |  |  |
| --- | --- | --- | --- | --- | --- | --- | --- | --- | --- | --- | --- | --- | --- | --- | --- |
| **Destination state** | 0 disease | 95% CI | | 1 disease | 95% CI | | Disability-free Multimorbidity | 95% CI | | Disabling Multimorbidity | 95% CI | | **Average** | 95% CI | |
| 0 disease | 9.9 | 9.1 | 10.8 | 0.0 | 0.0 | 0.0 | 0.0 | 0.0 | 0.0 | 0.0 | 0.0 | 0.0 | 1.4 | 1.3 | 1.5 |
| 1 disease | 7.0 | 6.5 | 7.5 | 9.3 | 8.7 | 9.8 | 0.0 | 0.0 | 0.0 | 0.0 | 0.0 | 0.0 | 3.4 | 3.2 | 3.6 |
| Disability-free Multimorbidity | 7.4 | 6.9 | 8.0 | 11.6 | 11.1 | 12.2 | 18.5 | 17.9 | 19.1 | 0.0 | 0.0 | 0.0 | 12.2 | 11.8 | 12.6 |
| Disabling Multimorbidity | 3.6 | 3.2 | 3.9 | 5.0 | 4.7 | 5.4 | 6.2 | 5.8 | 6.6 | 16.4 | 15.6 | 17.2 | 7.2 | 6.7 | 7.6 |
| Total | 27.9 | 27.4 | 28.4 | 25.9 | 25.5 | 26.4 | 24.7 | 24.2 | 25.2 | 16.4 | 15.6 | 17.2 | 24.1 | 23.7 | 24.6 |

# Section V: Life expectancy comparison with vital statistics and other studies

| **Data source** | **Index** | **Year** | **Male** | | | **Female** | | |
| --- | --- | --- | --- | --- | --- | --- | --- | --- |
|  |  |  | **Costa Rica** | **Mexico** | **United States** | **Costa Rica** | **Mexico** | **United States** |
| Our study | LE60  (95% CI) | 2005-2009 | 24.3  (22.9-25.8) |  |  | 25.1  (23.3-26.9) |  |  |
|  |  | 2012-2018 |  | 22.9  (21.8-24.0) |  |  | 25.4  (24.4-26.3) |  |
|  |  | 2004-2018 |  |  | 20.8  (20.5-21.2) |  |  | 23.1  (22.8-23.4) |
| **Vital statistics** |  |  |  |  |  |  |  |  |
| World Health Organization (2020) | LE60 | 2005 | 22.4 | 20.3 | 20.6 | 25.2 | 22.7 | 23.4 |
|  |  | 2010 | 22.5 | 20.3 | 21.5 | 25.4 | 22.7 | 24.2 |
|  |  | 2015 | 23.3 | 20.4 | 21.7 | 26.4 | 22.9 | 24.4 |
| United Nations Population Division (2022) | LE60 | 2005 | 21.3 | 20.0 | 20.6 | 24.0 | 22.3 | 23.7 |
|  |  | 2010 | 21.3 | 19.7 | 21.6 | 24.1 | 22.1 | 24.6 |
|  |  | 2015 | 21.2 | 19.7 | 21.9 | 24.3 | 22.3 | 24.8 |
| **Other studies** |  |  |  |  |  |  |  |  |
| Payne (2015) | LE65  (95% CI) | 2001-2003 |  | 18.4  (16.3-20.7) |  |  | 19.2  (17.4-21.5) |  |
|  |  | 2005-2009 | 19.0  (17.8-20.8) |  |  | 20.1  (18.8-21.9) |  |  |
|  |  | 2004-2010 |  |  | 18.1  (17.7-18.6) |  |  | 20.5  (20.1-20.9) |
| Mehta & Myrskylä (2017) | LE50 | 1998-2012 |  |  | 27.7  (26.9-29.0) |  |  | 31.4  (30.7-32.5) |
| Rosero-Bixby (2018) | LE60  (95% CI) | 2002-2011 |  | 21.9  (20.5-21.8) |  |  | 23.4  (22.8-24.0) |  |
|  |  | 2002-2012 | 21.9  (21.5-22.2) |  |  | 24.3  (23.9-24.8) |  |  |
| Rueda-Salazar (2021) | LE60  (95% CI) | 2005-2007 | 22.9  (17.9-26.4) |  |  | 26.2  (21.4-30.0) |  |  |

Source: Mehta & Myrskylä, 2017; Payne, 2018; Rosero-Bixby, 2018; Rueda-Salazar et al., 2021; United Nations Population Division, Department of Economic and Social Affairs, 2022; World Health Organization, 2020

# Section VI. Multimorbidity-free life expectancy, multimorbid life expectancy, and life expectancy at age 60


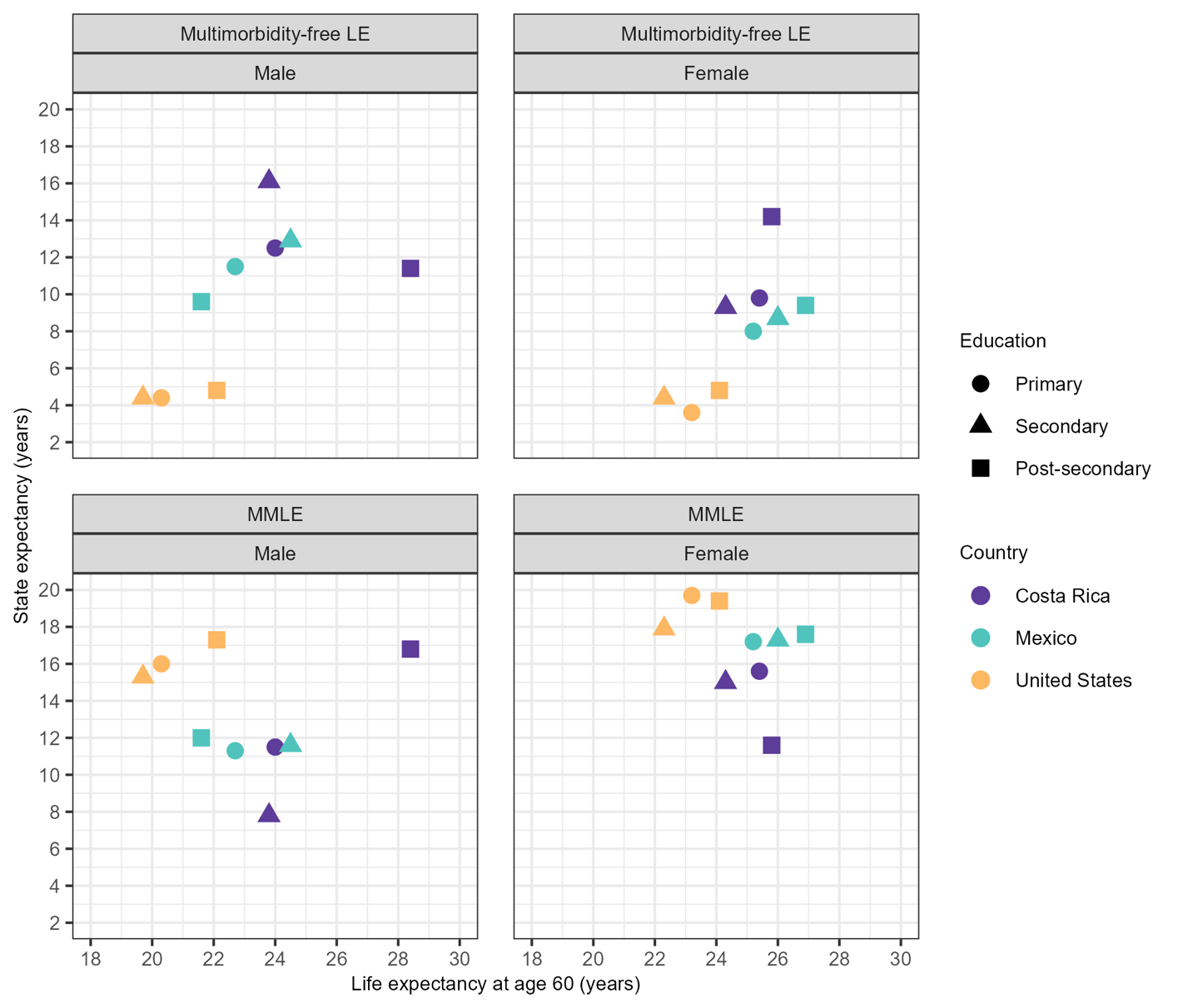


*Note.* LE: life expectancy, MMLE: Multimorbid life expectancy

# Section VII: Evidence for cumulative (dis)advantage

Sex difference in multimorbid life expectancy by education level in Costa Rica, Mexico, and the United States. Low education indicates an educational attainment of primary school or less, and high education indicates an educational attainment of post-secondary school.

| **Country** | **Low education** | | | **High education** | | |
| --- | --- | --- | --- | --- | --- | --- |
|  | Female MMLE | Male MMLE | Difference  (Female MMLE – Male MMLE) | Female MMLE | Male MMLE | Difference  (Female MMLE – Male MMLE) |
| Costa Rica | 15.6 | 11.5 | 4.1 | 11.6 | 16.8 | -5.2 |
| Mexico | 17.2 | 11.3 | 5.9 | 17.6 | 12.0 | 5.6 |
| United States | 19.7 | 16.0 | 3.7 | 19.4 | 17.3 | 2.1 |

*MMLE: Multimorbid life expectancy*

# Section VIII: Sensitivity analysis excluding hypertension from multimorbidity definition

| **Country** | **Sex** | **Destination state** | **Sensitivity analysis average expectancy** | **Lower 95% CI** | **Upper 95% CI** | **Main analysis average expectancy** | **Difference between sensitivity and main expectancies** |
| --- | --- | --- | --- | --- | --- | --- | --- |
| Costa Rica | Male | 0 disease | 10.9 | 10 | 11.7 | 6.2 | -4.7 |
|  |  | 1 disease | 8.7 | 7.9 | 9.5 | 6.6 | -2.1 |
|  |  | Disability-free multimorbidity | 2.5 | 1.8 | 3.2 | 6 | 3.5 |
|  |  | Disabling multimorbidity | 2.8 | 1.8 | 3.8 | 5.5 | 2.7 |
|  |  | Multimorbidity-free life expectancy | 19.6 | 17.9 | 21.2 | 12.8 | -6.8 |
|  |  | Multimorbid life expectancy | 5.3 | 3.6 | 7 | 11.5 | 6.2 |
|  |  | Life expectancy | 24.8 | 23.6 | 26 | 24.3 | -0.5 |
|  | Female | 0 disease | 7.5 | 6.7 | 8.3 | 2.5 | -5 |
|  |  | 1 disease | 9.3 | 8.3 | 10.3 | 7.5 | -1.8 |
|  |  | Disability-free multimorbidity | 3.1 | 2.4 | 3.7 | 6.6 | 3.5 |
|  |  | Disabling multimorbidity | 5.8 | 4.7 | 7 | 8.6 | 2.8 |
|  |  | Multimorbidity-free life expectancy | 16.8 | 15 | 18.6 | 10 | -6.8 |
|  |  | Multimorbid life expectancy | 8.9 | 7.1 | 10.7 | 15.2 | 6.3 |
|  |  | Life expectancy | 25.6 | 24 | 27.2 | 25.1 | -0.5 |
| Mexico | Male | 0 disease | 8.9 | 8 | 9.7 | 4.9 | -4 |
|  |  | 1 disease | 8.2 | 7.4 | 9 | 6.6 | -1.6 |
|  |  | Disability-free multimorbidity | 2.8 | 2.3 | 3.3 | 7 | 4.2 |
|  |  | Disabling multimorbidity | 2.9 | 2.2 | 3.7 | 4.4 | 1.5 |
|  |  | Multimorbidity-free life expectancy | 17.1 | 15.4 | 18.7 | 11.5 | -5.6 |
|  |  | Multimorbid life expectancy | 5.7 | 4.5 | 7 | 11.4 | 5.7 |
|  |  | Life expectancy | 22.8 | 21.8 | 23.9 | 22.9 | 0.1 |
|  | Female | 0 disease | 6.2 | 5.7 | 6.7 | 2.5 | -3.7 |
|  |  | 1 disease | 8.9 | 8.3 | 9.5 | 5.6 | -3.3 |
|  |  | Disability-free multimorbidity | 4.7 | 4.1 | 5.2 | 9.3 | 4.6 |
|  |  | Disabling multimorbidity | 5.5 | 4.6 | 6.3 | 7.9 | 2.4 |
|  |  | Multimorbidity-free life expectancy | 15.1 | 14 | 16.2 | 8.1 | -7 |
|  |  | Multimorbid life expectancy | 10.2 | 8.7 | 11.5 | 17.2 | 7 |
|  |  | Life expectancy | 25.3 | 24.3 | 26.3 | 25.4 | 0.1 |
| United States | Male | 0 disease | 2.9 | 2.8 | 3.1 | 1.4 | -1.5 |
|  |  | 1 disease | 5.2 | 5 | 5.4 | 3.2 | -2 |
|  |  | Disability-free multimorbidity | 8.8 | 8.5 | 9.1 | 11.9 | 3.1 |
|  |  | Disabling multimorbidity | 3.9 | 3.7 | 4.1 | 4.4 | 0.5 |
|  |  | Multimorbidity-free life expectancy | 8.1 | 7.8 | 8.5 | 4.6 | -3.5 |
|  |  | Multimorbid life expectancy | 12.7 | 12.2 | 13.2 | 16.3 | 3.6 |
|  |  | Life expectancy | 20.8 | 20.5 | 21.1 | 20.8 | 0 |
|  | Female | 0 disease | 2.4 | 2.3 | 2.5 | 1.3 | -1.1 |
|  |  | 1 disease | 6.3 | 6.1 | 6.5 | 3.3 | -3 |
|  |  | Disability-free multimorbidity | 8 | 7.7 | 8.2 | 11.1 | 3.1 |
|  |  | Disabling multimorbidity | 6.5 | 6.2 | 6.7 | 7.4 | 0.9 |
|  |  | MMFLE | 8.7 | 8.4 | 9 | 4.6 | -4.1 |
|  |  | MMLE | 14.5 | 13.9 | 14.9 | 18.5 | 4 |
|  |  | Total life expectancy | 23.1 | 22.8 | 23.4 | 23.1 | 0 |

Notes. CI: Confidence interval, MMLE: Multimorbid life expectancy.

# References

Mehta, N., & Myrskylä, M. (2017). The Population Health Benefits Of A Healthy Lifestyle: Life Expectancy Increased And Onset Of Disability Delayed. *Health Affairs*, *36*(8), 1495–1502. https://doi.org/10.1377/hlthaff.2016.1569

Payne, C. F. (2018). Aging in the Americas: Disability-free Life Expectancy Among Adults Aged 65 and Older in the United States, Costa Rica, Mexico, and Puerto Rico. *The Journals of Gerontology: Series B*, *73*(2), 337–348. https://doi.org/10.1093/geronb/gbv076

Rosero-Bixby, L. (2018). High life expectancy and reversed socioeconomic gradients of elderly people in Mexico and Costa Rica. *Demographic Research*, *38*(3), 95–108. https://doi.org/10.4054/DemRes.2018.38.3

Rueda-Salazar, S., Spijker, J., Devolder, D., & Albala, C. (2021). The contribution of social participation to differences in life expectancy and healthy years among the older population: A comparison between Chile, Costa Rica and Spain. *PLOS ONE*, *16*(3), e0248179. https://doi.org/10.1371/journal.pone.0248179

United Nations Population Division, Department of Economic and Social Affairs. (2022). *World Population Prospects: The 2022 Revision, custom data acquired via website*. https://population.un.org/wpp/

World Health Organization. (2020). *Global Health Observatory: Life expectancy at age 60*. World Health Organization. https://www.who.int/data/gho/data/indicators/indicator-details/GHO/life-expectancy-at-age-60-(years)
